# Transcriptomic responses of mixed cultures of ascomycete fungi to lignocellulose using dual RNA-seq reveal inter-species antagonism and limited beneficial effects on CAZyme expression

**DOI:** 10.1016/j.fgb.2016.04.005

**Published:** 2017-05

**Authors:** Paul Daly, Jolanda M. van Munster, Matthew Kokolski, Fei Sang, Martin J. Blythe, Sunir Malla, Juliana Velasco de Castro Oliveira, Gustavo H. Goldman, David B. Archer

**Affiliations:** a School of Life Sciences, University of Nottingham, University Park, Nottingham NG7 2RD, UK; b Deep Seq, Faculty of Medicine and Health Sciences, Queen’s Medical Centre, University of Nottingham, Nottingham NG7 2UH, UK; c Laboratório Nacional de Ciência e Tecnologia do Bioetanol (CTBE), Centro Nacional de Pesquisa em Energia e Materiais (CNPEM), Rua Giuseppe Máximo Scolfaro 10000, Campinas, São Paulo 13083-100, Brazil; d Faculdade de Ciências Farmacêuticas de Ribeirão Preto, Universidade de São Paulo, Avenida do Café, Ribeirão Preto, São Paulo 14040-903, Brazil

**Keywords:** An, *Aspergillus niger*, CAZy, carbohydrate active enzyme or protein, as found in the CAZy database, CAZyme, carbohydrate active enzyme, FPKM, uniquely mapped **F**ragments **P**er **K**ilobase of gene model per **M**illion uniquely mapped fragments, MWCO, molecular weight cut-off, *p_adj_*, *p* value from DESeq analysis with adjustment for multiple hypothesis testing, Pc, *Penicillium chrysogenum*, pNP-Ara, 4-Nitrophenyl-α-l-arabinofuranoside, pNP-α-Glc, 4-Nitrophenyl-α-d-glucopyranoside, pNP-β-Glc, 4-Nitrophenyl-β-d-glucopyranoside, pNP-Cel, 4-Nitrophenyl-β-d-cellobioside, pNP-Xyl, 4-Nitrophenyl-β-d-xylopyranoside, Tr, *Trichoderma reesei*, *Aspergillus niger*, *Trichoderma reesei*, *Penicillium chrysogenum*, Lignocellulose, Mixed cultures, Dual RNA-seq, Competition

## Abstract

Gaining new knowledge through fungal monoculture responses to lignocellulose is a widely used approach that can lead to better cocktails for lignocellulose saccharification (the enzymatic release of sugars which are subsequently used to make biofuels). However, responses in lignocellulose mixed cultures are rarely studied in the same detail even though in nature fungi often degrade lignocellulose as mixed communities.

Using a dual RNA-seq approach, we describe the first study of the transcriptional responses of wild-type strains of *Aspergillus niger*, *Trichoderma reesei* and *Penicillium chrysogenum* in two and three mixed species shake-flask cultures with wheat straw.

Based on quantification of species-specific rRNA, a set of conditions was identified where mixed cultures could be sampled so as to obtain sufficient RNA-seq reads for analysis from each species. The number of differentially-expressed genes varied from a couple of thousand to fewer than one hundred. The proportion of carbohydrate active enzyme (CAZy) encoding transcripts was lower in the majority of the mixed cultures compared to the respective straw monocultures. A small subset of *P. chrysogenum* CAZy genes showed five to ten-fold significantly increased transcript abundance in a two-species mixed culture with *T. reesei*. However, a substantial number of *T. reesei* CAZy transcripts showed reduced abundance in mixed cultures. The highly induced genes in mixed cultures indicated that fungal antagonism was a major part of the mixed cultures. In line with this, secondary metabolite producing gene clusters showed increased transcript abundance in mixed cultures and also mixed cultures with *T. reesei* led to a decrease in the mycelial biomass of *A. niger*. Significantly higher monomeric sugar release from straw was only measured using a minority of the mixed culture filtrates and there was no overall improvement.

This study demonstrates fungal interaction with changes in transcripts, enzyme activities and biomass in the mixed cultures and whilst there were minor beneficial effects for CAZy transcripts and activities, the competitive interaction between *T. reesei* and the other fungi was the most prominent feature of this study.

## Highlights

• First genome-wide transcriptional response in fungal mixed species straw cultures.
• In mixed cultures, rRNA abundance was used to predict RNA-seq read abundance.
• Subset of *P. chrysogenum* CAZy with mixed cultures increased abundance pattern.
• Lack of overall higher CAZy transcripts/activities due to inter-species antagonism.
• Induction of secondary metabolite producing gene clusters in mixed cultures.

## Graphical abstract

**Figure:**
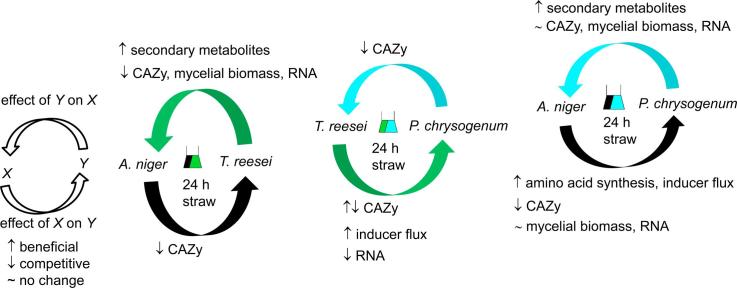

## 1 Introduction

Lignocellulose is an abundant raw material that has the potential to be a cost-effective source of liquid biofuels. A key limitation of the process is the saccharification stage which requires costly enzymes (Klein-Marcuschamer et al., 2012). The enzymes required are lignocellulose degrading carbohydrate active enzymes (CAZymes) which include cellulases, hemicellulases and accessory activities (van den Brink and de Vries, 2011). One approach to reduce the costs is to exploit a better understanding of the fungal responses to lignocellulose and understanding the responses of fungi in mixed cultures forms part of this approach. Fungal mixed cultures are mixtures of two or more individual fungal species or strains. Part of the rationale for using mixed cultures to degrade lignocellulose is that different fungal species can be found in the same lignocellulose-containing ecological niche such as a hollow tree stump (Tian et al., 2010) or leaves (Das et al., 2007). The inter-species fungal interaction are most likely to be based on antagonism and competition (Boddy, 2000) although co-operative interactions are also possible that could benefit the saccharification of lignocellulose. Enzymes from lignocellulose-containing mixed cultures could be advantageous over combining enzymes from monocultures because of enzymatic and gene regulatory reasons. Unique activities from one fungus could expose sugar inducers from the lignocellulose that the enzyme activities of the other fungus might have been incapable of exposing such as in a complex pectin structure thus resulting in a more complete induction of CAZymes in that other fungus. We explored the interactions of wild-type strains of *Aspergillus niger*, *Trichoderma reesei* and *Penicillium chrysogenum* in two and three mixed species shake-flask cultures with wheat straw using a range of approaches including dual RNA-seq.

Genome-wide transcriptomic studies could elucidate how transcript abundance changes in a mixed culture compared to monocultures. Arfi et al. (2013) studied, using a standard complex laboratory medium, mixed cultures with basidiomycetes that competed. RNA-seq analysis was performed only on the out-competing fungus from the mixed culture, *Pycnoporus coccineus*, showing that genes involved in detoxification of secondary metabolites had higher transcript abundance compared to the monoculture (Arfi et al., 2013). Jonkers et al. (2012) analysed mixed cultures of two plant-infecting fungi but their use of microarrays did not facilitate an accurate measure of total RNA from each species in their mixed cultures. To our knowledge, there is no literature on the genome-wide transcriptional responses of mixed cultures to lignocellulose degradation or of the antagonistic responses of different species when exposed to lignocellulose as mixed cultures.

There is substantial literature on the effects at the enzymatic level of mixed cultures and in particular with *T. reesei*, which is one of the most studied fungal species in mixed cultures (Ahamed and Vermette, 2008, Duff et al., 1987, Kolasa et al., 2014) as well as two studies that combined ascomycetes and basidiomycetes (Hu et al., 2011, Ma and Ruan, 2015). Culture filtrates from a mixed culture of *T. reesei* and a basidiomycete *Coprinus comatus* had a clear synergistic effect on saccharification compared to that of the monoculture filtrates, likely in part due to the de-lignifying activities of the basidiomycete (Ma and Ruan, 2015). Several studies show beneficial effects of mixed cultures for enzymatic activities but those studies did not culture the fungi with lignocellulose or did not perform saccharification assays with lignocellulose. One of the earlier studies showed there were beneficial effects when *T. reesei* was cultured with *Aspergillus* spp. where *T. reesei*, which is deficient in secreting β-glucosidases (or transcribing genes that encode β-glucosidases), was complemented by the secreted activities of *A. niger* (Duff et al., 1987). In the study of Hu et al. (2011) beneficial effects on enzymatic activities relevant to lignocellulose degradation were highest in mixed cultures of *Aspergillus oryzae* and *Phanerochaete chrysosporium*. Ahamed and Vermette (2008) reported a beneficial effect with higher volumetric filter paper activity in a mixed culture of *A. niger* and *T. reesei*, but not a higher filter paper activity per amount of fungal biomass compared to an *A. niger* monoculture.

There are several genome-wide transcriptomic studies of the response of monocultures of fungi to lignocellulose. These studies are in *A. niger* (de Souza et al., 2011, Delmas et al., 2012, Pullan et al., 2014, van Munster et al., 2014), *Aspergillus nidulans* (Brown et al., 2013, Coradetti et al., 2013), *T. reesei* (Bischof et al., 2013, Foreman et al., 2003, Hakkinen et al., 2012, Ries et al., 2013), *Neurospora crassa* (Benz et al., 2014, Tian et al., 2009) and *Myceliopthora thermophila* (Kolbusz et al., 2014). The regulatory basis for how fungi respond to lignocellulose consists of activating and repressing transcription factors that themselves respond to sugar molecules derived from the lignocellulose (for reviews see Daly et al., 2016, Glass et al., 2013, Kowalczyk et al., 2014). In *A. niger*, the main activating transcription factor is XlnR which is activated by xylose (van Peij et al., 1998). In *T. reesei* the orthologous transcription factor XYR1 is also the main activating transcription factor but it is responsive to other sugars as well as xylose, such as cellobiose and sophorose (Stricker et al., 2008, Stricker et al., 2006).

The three species chosen for this study were ascomycetes from three different genera; *A. niger*, *T. reesei* and *P. chrysogenum*. *T. reesei* is the dominant CAZyme producer used by industry (Martinez et al., 2008). *A. niger*, also a CAZyme producer, is an extensively studied species with regard to regulation of biomass-degrading CAZy genes and has a large repertoire of CAZymes (Andersen et al., 2011, Kowalczyk et al., 2014, Pel et al., 2007). *P. chrysogenum* is best known as a producer of β-lactam antibiotics, but *Penicillium* species are also enzyme producers and degrade lignocellulose in nature (Chávez et al., 2006, Gusakov and Sinitsyn, 2012) and *P. chrysogenum* is one of the *Penicillium* species that has a sequenced and annotated genome (van den Berg et al., 2008). Wild-type strains rather than industrial production strains were used so to be more similar to the sensing of the lignocellulose in nature. Industrial strains such as *T. reesei* RUT-C30 are modified in their regulatory circuits involved in carbon sensing as well as having an increased ability to secrete protein (Peterson and Nevalainen, 2012). Differences in enzymatic activities provide part of the rationale for combining fungal species in mixed cultures with lignocellulose and *T. reesei* lacks important activities. The absence of genes encoding pectin methylesterase activity and feruloyl esterase activity in *T. reesei* was noted by Martinez et al. (2008). Akel et al. (2009) also noted the absence of endo-arabinases in *T. reesei.*

At the genome-wide level, dual RNA-seq or simultaneous RNA-seq is a technique that allows the quantification of transcripts from multiple organisms simultaneously and the technique is primarily applied to host pathogen interactions (Westermann et al., 2012). Dual RNA-seq has the potential to be applied to mixed species fungal cultures, provided that there are sufficient nucleotide differences of the RNA-seq reads sequenced from each fungus and that sufficient reads can be obtained from each fungus in a mixed culture.

We will describe differences in gene content and enzymatic activities between the three fungal species that informed our experimental conditions for subsequent dual RNA-seq analysis. We used a method, which could be widely applicable, to quantify species-specific rRNA in mixed cultures to predict the number of RNA-seq reads that could be obtained. Subsequently, we will describe the limited beneficial effects and more widespread antagonistic effects on mycelial biomass, transcript abundance and enzyme activities of combining the three fungi in two and three species mixed cultures.

## 2 Materials and methods

### 2.1 Strains and growth conditions on solid media

To obtain spores, *A. niger* N402 was cultured on potato dextrose agar (PDA) (Oxoid) slopes for ∼5 days at 28 °C, *T. reesei* QM6a was cultured on PDA plates for between 5 and 10 days at 28 °C and *P. chrysogenum* Wisconsin54-1255 was cultured on *Aspergillus* minimal agar plates at pH 6.5 with 1% (w/v) xylan (Sigma Cat# X4252) as carbon source and NH_4_Cl as nitrogen source for ∼4 weeks at 28 °C. The *Aspergillus* minimal media used in agar plates was otherwise as described in Delmas et al. (2012).

### 2.2 Mono and mixed species liquid cultures

#### 2.2.1 Preparation of CCMM and washed straw solutions

Co-culture minimal media (CCMM) composition was based on minimal medium for *A. niger* (Delmas et al., 2012), *T. reesei* (Ries et al., 2013) and *P. chrysogenum* (Haas et al., 1997), had a pH of ∼4.5 and contained 5 g (NH_4_)_2_SO_4_ l^−1^, 0.6 g MgSO_4_ l^−1^, 0.79 g CaCl_2_·2H_2_O l^−1^, 0.52 g KCl l^−1^, 3 g KH_2_PO_4_ l^−1^ and the trace elements 63.7 mg EDTA·Na_2_·2H_2_O l^−1^, 1.4 mg ZnSO_4_·7H_2_O l^−1^, 50 μg H_3_BO_3_ l^−1^_,_ 5 mg FeSO_4_·7H_2_O l^−1^, 160 μg FePO_4_ l^−1^_,_ 3.7 mg CoCl_2_·6H_2_O l^−1^, 250 μg CuSO_4_·5H_2_O l^−1^, 2.04 mg MnSO_4_·4H_2_O l^−1^, 160 μg NaMoO_4_·2H_2_O l^−1^, 8 μg Na_2_B_4_O_7_·10H_2_O l^−1^, 1 mg Fe(NH_4_)_2_(SO_4_)_2_·6H_2_O l^−1^. Trace elements were prepared separately as a 1000 × stock solution by dissolving the components at a pH of >6, adjusting the pH to 4 and sterilisation through a 0.2 μm filter. Wheat straw was ball-milled as described previously (Delmas et al., 2012), re-suspended in water at 1% (w/v) concentration and mixed. 500 ml of this solution was filtered through 0.22 μM Stericup filtration units (Millipore) (PES membrane) and washed twice with 500 ml water. The washed straw was then re-suspended in 500 ml of CCMM and autoclaved at 117 °C.

#### 2.2.2 Preparation of mono and mixed species liquid cultures

Spores were harvested using a 0.1% (v/v) Tween 20 solution from solid media for each of the species. Three biological replicate groups (A, B and C) of monoculture glucose pre-cultures for each species were inoculated in 250 ml shake-flasks containing 100 ml of 1% (w/v) glucose in CCMM at the following spore concentrations and incubation times at 150 RPM and 28 °C; *A. niger* 1 · 10^6^ ml^−1^ for ∼36 h, *T. reesei* 1 · 10^5^ ml^−1^ for ∼36 h and *P. chrysogenum* 5 · 10^5^ ml^−1^ for ∼48 h. For *A. niger* and *P. chrysogenum*, the mycelia from shake-flasks from a glucose monoculture replicate group were pooled by filtering through Miracloth (Calbiochem) then washed with ∼100 ml of CCMM. For *T. reesei*, the mycelia from each individual shake-flask from a glucose monoculture replicate group was filtered through Miracloth separately and washed with ∼100 ml of CCMM then pooled to make the replicate group of mycelia of sufficient amount for subsequent transfer. 0.75 g wet weight of mycelia from a replicate glucose culture group was transferred to the respective straw replicate culture, in 250 ml shake-flasks containing 100 ml of 1% (w/v) washed wheat straw to make either monocultures or two or three species mixed cultures and incubated for various lengths of time at 28 °C.

### 2.3 RNA isolation and cDNA synthesis

Mycelia were ground under liquid nitrogen using a mortar and pestle, then the RNA was purified using the Plant/Fungi total RNA Purification Kit (Norgen Biotek, Canada) including the on-column DNase treatment step. The concentration and quality of RNA for each sample was determined by UV spectrometry (Nanodrop ND-1000 spectrophotometer). cDNA was synthesised as described previously (Delmas et al., 2012) except that 50 ng of Random Primer oligonucleotides (Invitrogen) for each 20 μl cDNA synthesis reaction were used.

### 2.4 Mycelial biomass estimation using genomic DNA

The solids from the straw cultures were collected by centrifugation and were freeze-dried. Between 75 mg and 120 mg of the freeze-dried solids from the straw cultures was used for gDNA extraction. The gDNA was extracted in 2 ml tubes with 1.5 ml extraction buffer (50 mM Tris-HCl (pH 7.5), 10 mM EDTA, 50 mM NaCl and 1% w/v SDS) by incubating at 65 °C for 1 h with continuous mixing followed by separation with phenol:chloroform:isoamylalcohol (25:24:1, v/v/v) using PhaseLock tubes (5 Prime). After precipitation, the gDNA was re-suspended in TE and RNase treated followed by a second precipitation and wash to remove the degraded RNA. The amount of extracted gDNA was converted to biomass (dry-weight) using standard curves of the qPCR signal intensity from amplification of gDNA extracted from between 2 mg and 50 mg of freeze-dried mycelia from each species from glucose monocultures. When gDNA was extracted from mycelia from glucose cultures, 50 mg of straw was also added to the extractions to control for any effect the straw could have on the extraction efficiency. gDNA was quantified using a qPCR assay as described below. In this assay, a 1/500 dilution of the gDNA (to ∼0.1 ng) was made from standards and samples to avoid any inhibitory effects on the polymerase. The total mycelial biomass in the shake-flask was estimated by factoring in the proportion of the sample used for gDNA extraction in relation to the total sample recovered from the shake-flask. The primer and probe sets are described in Supplementary Table S1.

### 2.5 Quantification of 5.8S rRNA for RNA abundance measurement or single copy region of gDNA for biomass prediction from each fungus in mixed cultures

#### 2.5.1 Primer and probe design

Primer and dual-labelled hydrolysis Taqman-type probes (Supplementary Table S1) to amplify rRNA or other sequences from the cDNA or gDNA from each fungus were designed using two software tools; OligoArchitect (Sigma) and Beacon Designer 8 (PREMIER Biosoft). The SNP discriminating function in Beacon Designer was used to design probes that discriminated between the 5.8S rRNA regions from each fungus. For designing primers and probes for the 5.8S rRNA, the following sequences were used: *A. niger*: GenBank JX535496.1, *T. reesei*: GenBank Z31016.1 and *P. chrysogenum*: sequence from ATCC28089 obtained from http://www.lgcstandards-atcc.org/. The dual-labelled probes (Sigma) contained several locked nucleic acid (LNA) bases (Supplementary Table S1). The reporter labelled on the 5′ end was 6′-FAM and the quencher labelled on the 3′ end was BHQ-1. For gDNA, regions of each genome that were single copy were used (See Supplementary Table S1 for further information).

#### 2.5.2 Preparation of plasmid standards for quantification with qPCR

To generate standards for quantification, the regions containing the rRNA were amplified from gDNA using primers listed in Supplementary Table S1. The amplified products were cloned into pBluescript and the plasmids purified from *E. coli* and sequenced using standard procedures. The copy number of target sequence for amplification from a quantity of plasmid was determined with http://www.thermoscientificbio.com/webtools/copynumber/. Standard curves were made with ∼10 5-fold dilutions of the linearised plasmids starting with 50 M target copies. The plasmids were also used to determine the specificity of the primer and probe combinations for their intended targets as opposed to the off-target from the other species.

#### 2.5.3 Assay conditions for quantification and normalisation

The FastStart Universal Probe Master Mix (Roche) was used according to the manufacturer’s instructions. The reaction set-up used 10 μl reactions with 1 μl of the cDNA (diluted 1/100 for rRNA quantification), gDNA or plasmid templates. The primer and probe concentrations used are indicated in Supplementary Table S1. The 7500 Real-time machine (Applied Biosystems) was used with the cycling conditions of 95 °C for 10 min followed by 40 cycles of 95 °C for 15 s and 60 °C for 1 min. To normalise the transcript levels from the protein encoding genes, the species-specific 5.8S rRNA abundance was used.

### 2.6 Genome and other annotation resources used

The following genomes and annotation sets were used. For *A. niger* the ATCC1015 strain sequence and annotation (Aspni7) (Andersen et al., 2011) from the JGI (http://genome.jgi-psf.org/Aspni7/Aspni7.home.html), for *T. reesei* the QM6a sequence and annotation (Trire2) (Martinez et al., 2008) from the JGI (http://genome.jgi-psf.org/Trire2/Trire2.home.html) and for *P. chrysogenum* the Wisconsin54-1255 strain sequence and annotation (PenchWisc1_1) (van den Berg et al., 2008) deposited at the JGI (http://genome.jgi.doe.gov/PenchWisc1_1/PenchWisc1_1.home.html). For *P. chrysogenum*, the CAZy annotations were downloaded from the CAZy database (Lombard et al., 2014) page http://www.cazy.org/e816.html on 21st August 2015. For *T. reesei*, the CAZy annotations from Hakkinen et al. (2012) were combined with those provided by Bernard Henrissat (personal communication) for Trire2 so as to annotate the maximum number of genes with CAZy annotations and also include annotation for Auxiliary Activity (AA) families which Hakkinen et al. (2012) did not annotate. For *A. niger*, the CAZy annotations provided by Bernard Henrissat (personal communication) for Aspni7 were used and supplemented with CAZy annotations from CBS513.88 strain where the orthologue was present in Aspni7 but had not been annotated with a CAZy annotation. The signal peptide predictions and InterPro annotations downloaded from the databases at the JGI were used. For the complete lists of annotations used for each species, see Supplementary Table S2.

### 2.7 Bioinformatic analysis to identify orthologous and non-orthologous proteins

Orthologous and non-orthologous proteins were identified using the Orthofinder orthogroup inference algorithm (Emms and Kelly, 2015). For this analysis the following protein fasta files were used from the JGI: Aspni7 – Aspni7_GeneCatalog_proteins_20131226.aa.fasta (11,910 proteins), Trire2 – TreeseiV2_FrozenGeneCatalog20081022.proteins.fasta (9,143 proteins) and Penchwisc1_1 – PenchWisc1_1_GeneCatalog_proteins_20140114.aa.fasta (13,671 proteins). In Supplementary Table S2, the orthogroups for each protein from each species are listed along with the species composition of the orthogroup.

### 2.8 Dual RNA-seq sequencing, read mapping and RNA-seq statistical analysis

Total RNA was measured using Qubit RNA BR assay kit (Life technologies, Q10210). 1 μg of Total RNA was used for enrichment of mRNA using NEBNext Poly(A) mRNA Magnetic Isolation Module (NEB, E7490). Illumina stranded whole transcriptome sequencing libraries were prepared using NEBNext Ultra Directional RNA library prep kit for Illumina (NEB, E7420S). Library QC was performed using bioanalyser HS kit (Agilent biotechnologies, 5067-4626). Libraries were quantified using qPCR (Kapa Biosystems, KK4824), pooled at desired concentrations, denatured and loaded for sequencing according to manufacturer’s instructions. Sequencing was done on Illumina NextSeq500 sequencing platform using 2 high output runs to generate 2 × 75 bp reads. Sufficient sequencing was done to obtain ∼30 M reads from each monoculture replicate and ∼100 M reads from each mixed culture replicate.

A filtering pipeline was used to filter reads with low sequencing quality score as well as reads aligned to adaptor sequences. First, raw reads were trimmed against adaptors using Scythe (https://github.com/vsbuffalo/scythe). Then reads were quality trimmed using Sickle (https://github.com/najoshi/sickle). For all samples, reads that passed the filters were mapped using the tophat mapping tool (https://ccb.jhu.edu/software/tophat/index.shtml) onto a composite reference genome that consisted of the genomes of each of the three species. The composite genome was made by concatenating into a single file the fasta files made of the sequences for the chromosomes from Aspni7, Trire2 and Penchwisc1_1 (where repeat regions had been masked). The use of this composite genome facilitated the exclusion from subsequent analysis of reads that were not unique to a particular species as these reads would not be uniquely mapped in the composite genome. Uniquely mapped read counts (at the MAPQ20 threshold) for each gene were calculated using ‘htseq-count’ (http://www-huber.embl.de/users/anders/HTSeq/doc/count.html). Read counts were calculated for each species separately using a genome annotation file for each species that contained the known gene exon co-ordinates for the genes for that species. The genome annotation files used were as follows: Aspni7 – Aspni7_GeneCatalog_genes_20131226.gff, Trire2 – TreeseiV2_FrozenGeneCatalog20081022.gff and Penchwisc1_1 – PenchWisc1_1_GeneCatalog_genes_20140114.gff. Principal component analysis (PCA) was used to indicate if the biological replicates were sufficiently similar for subsequent statistical analysis. The read counts of genes values for each species were subsequently used for statistical analysis using DESeq2 version 1.10.1 (Love et al., 2014). FPKM normalised read counts were defined originally by Mortazavi et al. (2008). In our study, an FPKM value for a gene from a sample is the number of uniquely mapped (to the composite genome) fragments (counts) per kilobase of gene model per million uniquely mapped (to the composite genome) fragments (counts) onto gene models from that species. The RNA-seq reads from this project were submitted to the GEO database (GEO accession GSE81187).

### 2.9 Hierarchical clustering and GO enrichment analysis

Clustering was performed using the GenePattern software platform http://www.broadinstitute.org/cancer/software/genepattern (Reich et al., 2006) where the Hierarchical Clustering module was used (de Hoon et al., 2004, Eisen et al., 1998). The dataset used for the clustering analysis consisted of the FPKM values of genes with a particular annotation which had significantly different changes in expression from the respective glucose control (*p*_adj_ < 0.05) and an FPKM ⩾ 1 in at least one of the straw conditions. The FPKM values were log transformed within the Hierarchical Clustering module. The ‘uncentered correlation’ option, which takes the magnitude of the values into account, was used as the distance measure followed by pairwise complete-linkage as the clustering method. The clustering results were visualised with the Hierarchical Clustering Viewer in GenePattern.

GOEAST GO enrichment tool was used (Zheng and Wang, 2008) by selecting the customised analysis tool with the default advanced parameter settings. The GO annotations were obtained from the JGI website for the respective species as described in other section. Lists of differentially expressed genes were analysed with the following cut-offs: ⩾1 FPKM value in one of the two conditions (which condition depended on whether genes with increased or reduced transcript abundance were being analysed) and ⩾2-fold changes (DESeq counts) in transcript abundance as well as a *p*_adj_ value < 0.05.

### 2.10 Enzymatic activity measurements using pNP-linked carbohydrate substrates

Culture filtrates from either two or three flask cultures were assayed using 4-Nitrophenyl-β-d-cellobioside (pNP-Cel), 4-Nitrophenyl-α-l-arabinofuranoside (pNP-Ara), 4-Nitrophenyl-β-d-glucopyranoside (pNP-β-Glc), 4-Nitrophenyl-β-d-xylopyranoside (pNP-Xyl) and 4-Nitrophenyl α-d-galactopyranoside (pNP-α-Gal) (all from Sigma). With assay conditions that gave a linear response between time and pNP release, up to 113 μl of the culture filtrates were assayed in a total volume of 130 μl with 2.5 mM final concentration of the substrate in 50 mM sodium acetate pH 5.0. The reaction mixtures were incubated at 37 °C for 3 h with shaking, and then stopped with 130 μl of 1 M sodium carbonate before the absorbance was measured at 405 nm with a plate reader (Thermo). The enzyme activity was expressed in ρmoles pNP per minute per μl of culture filtrate assayed (ρmol pNP · (min · μl)^−1^).

### 2.11 Concentration and quantification of proteins and PAGE gel analysis

Proteins in the culture filtrates were concentrated by 30–50-fold using 5000 kDa MWCO Vivaspin columns (GE Life Sciences) by centrifugation at 3000*g* at 4 °C. Proteins were quantified with Bradford assay (Sigma) using BSA as standard. The proteins in the concentrated culture filtrates were precipitated with TCA, followed by acetone washes and the pellets were re-solubilised in 25 μl of 8 M urea before diluting the urea concentration to 2 M with water. The BSA used in the standard curve was also precipitated in the same manner as the proteins from the culture filtrates. PAGE gels were run as described previously (Delmas et al., 2012) and visualised by following a silver staining method (Yan et al., 2000). A pre-stained protein ladder was used (NEB, cat# P7712).

### 2.12 Enzymatic activity assays with polysaccharides (saccharification assays)

Two different sets of conditions were used for the saccharification assays in this study. One set of conditions was used to predict the flux of sugars in the 24 h cultures analysed by RNA-seq and used the same media and incubation temperature as for culturing the fungi. The other set of conditions was used to determine whether the enzymes could have any industrial applications and used a buffer and incubation temperature commonly used for saccharification assays (referred to as standard-type saccharification reactions).

The saccharification assays to quantify glucose, xylose, arabinose and galactose (the latter three are inducers of CAZymes) released from straw so as to predict the flux of sugars in the 24 h cultures were performed in 0.5 ml tubes in a total volume of 450 μl with 15 mg of washed wheat straw, volumes of the concentrated 24 h culture filtrates equivalent to 1.5 ml of the original culture filtrates, CCMM and sodium azide at a 0.02% w/v final concentration. Reactions were incubated for 6 h at 28 °C with moderate shaking. The monosaccharides in the heat inactivated reactions were quantified with HPAEC-PAD.

Standard-type saccharification reactions were performed in 1.5 ml tubes in a total volume of 900 μl with 30 mg of wheat straw, volumes of each of the concentrated 4 d culture filtrates equivalent to 4.5 ml of the original culture filtrates, 50 mM citrate buffer pH 4.8 and sodium azide at a 0.02% w/v final concentration. Reactions were incubated for 72 h at 50 °C with moderate shaking. At various time points, aliquots of the hydrolysate were sampled and the enzymes were inactivated by heating at 100 °C for 5 min followed by centrifugation to pellet the solids. The total reducing sugars and other reducing end groups were quantified with the DNS assay (Dubois et al., 1956) using glucose as a standard. The sugars measured were corrected for the reducing end groups present in the straw and filtrate controls.

### 2.13 Measurement of monosaccharides in culture filtrates and saccharification hydrolysates

High-performance anion exchange chromatography with pulsed amperometric detection (HPAEC-PAD) with a Dionex ICS-3000 Ion Chromatography System (Dionex, UK) using a CarboPac PA20 column with a 10 mM NaOH isocratic system at a working flow rate of 0.5 ml min^−1^ at 30 °C. Glucose, xylose, arabinose and galactose were used as standards.

### 2.14 Statistical analyses

For data other than the RNA-seq data (where DESeq2 was used and is described elsewhere), GenStat (VSN International) was used to perform ANOVA analysis with Tukey’s test as a post-hoc test. The squared Pearson correlation coefficient (r^2^) was calculated by Excel.

## 3 Results and discussion

Strategies for fungal degradation of lignocellulose can vary depending on gene content, transcript abundance and secretion of enzymes with varying levels of activity; for example the *A. niger* genome encodes a more diverse array of hemicellulose and pectin degrading enzymes than *T. reesei* (Zhao et al., 2014). The lignocellulose degradation strategy of *P. chrysogenum* has not been substantially studied previously. We explored the differences in genome content of these fungi and the generation of enzymatic activities in response to straw (as well as their transcriptional response to straw degradation) before combining these fungi in mixed cultures for dual RNA-seq analysis.

### 3.1 Comparison of CAZy gene contents of *A. niger*, *T. reesei* and *P. chrysogenum* using orthologue analysis

CAZy families provide a useful framework to compare the lignocellulose saccharifying potential of fungal species, although a family can include multiple proteins and enzymatic activities so an orthologue analysis was used to further dissect the differences within CAZy families. Non-orthologous proteins in a CAZy family are more likely to encode for different activities. Previous analyses comparing CAZy gene contents of the three species are available such as the large scale study of Zhao et al. (2014) or the study of Liu et al. (2013) but neither included an orthologue analysis as part of their CAZy analysis. We used Orthofinder (Emms and Kelly, 2015) to analyse the full protein complements from each species (Supplementary Table S2) but will focus here on the CAZy gene contents of the three species by comparing the CAZy families (GH, CE, PL and AA) (Supplementary Table S3A–D). There were CAZy families that were present in only one of the three species but these did not confer any unique enzymatic activities relevant to lignocellulose saccharification. However, there were CAZy families that were present in *A. niger* and *P. chrysogenum* but not in *T. reesei* that conferred unique activities relevant to lignocellulose saccharification. The proteins in these CAZy families were encoded by orthologous genes indicating that the activities that *A. niger* and *P. chrysogenum* (but not *T. reesei*) had were likely the same activities. These were the CE8 and CE12 families whose proteins encode for pectin methylesterase and rhamnose acetylesterase activities respectively, the PL1 and PL4 families that encode for pectate lyase activity, feruloyl esterases from the CE1 family and endo-arabinases from the GH43 family. In summary, the genomes of *A. niger* and *P. chrysogenum* provide a broader range of enzymes for pectin degradation when compared to *T. reesei.* Whilst the unique relevant activities were few, other differences in how orthologous genes are regulated and how this could change in mixed cultures provided further rationale for investigating combinations of these three fungi.

### 3.2 Determination of appropriate conditions for dual RNA-seq experiment

To investigate differences in the responses of the monocultures to straw that would indicate whether combining the fungal species in mixed cultures could be beneficial; a time-course experiment was performed to measure transcript abundance of selected genes and various exo-acting activities from monocultures. Subsequently, saccharification assays were performed using culture filtrates from mono and mixed cultures. qPCR based methods were used to estimate total RNA and mycelia biomass from each fungus in a mixed culture. The purpose of estimating total RNA from each fungus was so as to predict whether the conditions chosen for mixed cultures would likely lead to sufficient RNA-seq reads from each fungus in a mixed culture.

#### 3.2.1 Measurement of transcript abundance and enzyme activities in straw monocultures

Transcript abundance changes were investigated through the orthologous genes encoding the reducing-end-acting GH7 cellobiohydrolases encoded by *A. niger cbhA* and *cbhB*, *P. chrysogenum Pc18g05490* and *Pc20g01970* and *T. reesei cbh1.* The respective genes all had increased transcript abundance in each species relatively early by 6 h after transfer to straw (Supplementary Figure S1). The temporal increase in transcript abundance of *A. niger cbhA*, *cbhB* and the *P. chrysogenum* orthologues was more incremental than *T. reesei cbh1* which had a larger fold change in transcript abundance between the glucose control and 3 h (the earliest straw culture time point assayed) than between 3 h and 6 h.

**Fig. 1:**
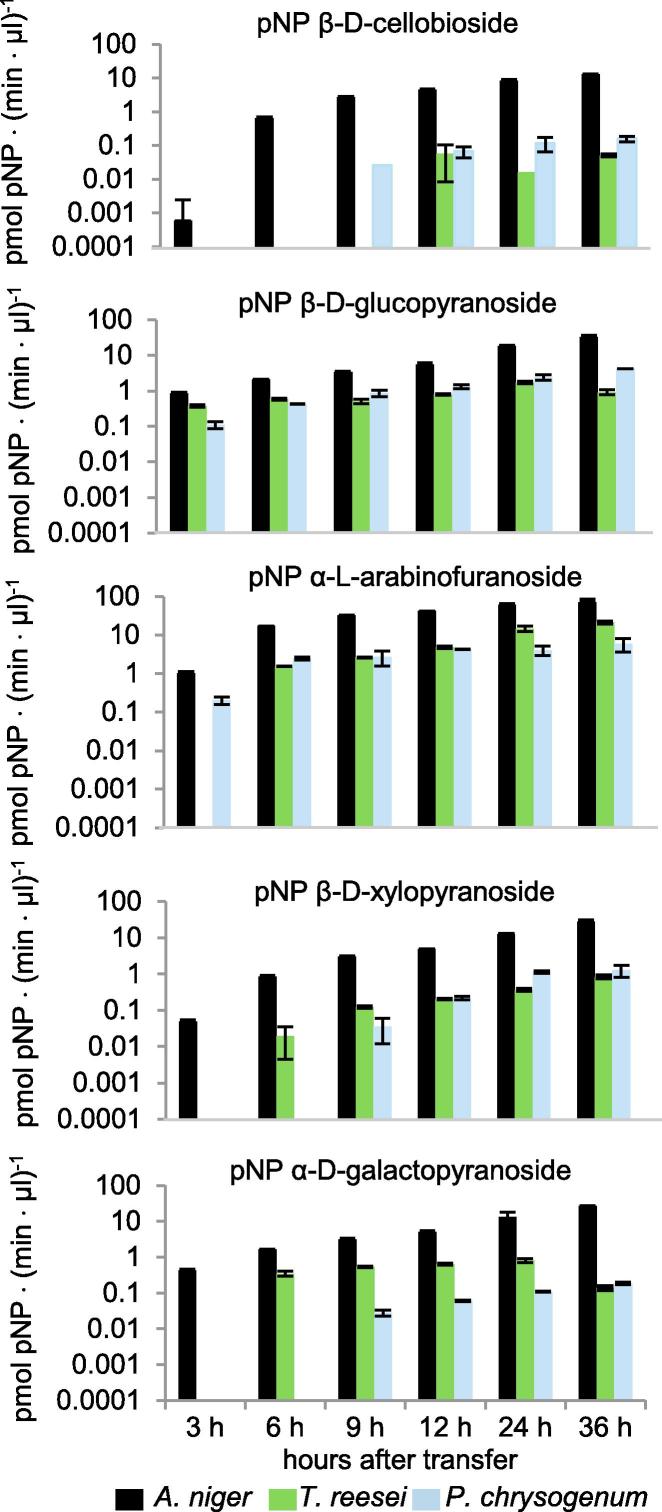
Activities towards pNP substrates from monoculture time-courses of *A. niger*, *T. reesei* or *P. chrysogenum*. Here activities from up to a maximum of 113 μl of unconcentrated culture filtrates were assayed. The error bars in these graphs represent the standard errors from two replicate flasks.

Activities were measured from the culture filtrates towards *para*-nitrophenol linked carbohydrate substrates. *A. niger* culture filtrates generally had more enzyme activity than either *T. reesei* or *P. chrysogenum* per equivalent volumes (Fig. 1). The extent to which the activity from the *A. niger* cultures was greater than from the cultures of either of the other fungi varied depending on the activity assayed. The levels of α-l-arabinofuranosidase activity from *T. reesei* filtrates were closer to the *A. niger* levels than was the case for the other four activities. For *P. chrysogenum*, the levels of β-glucosidase activity from the filtrates were closer to the *A. niger* levels than for the other four activities.

Based primarily on the enzymatic activities, the 24 h time point was considered appropriate to sample for mixed cultures. As well as the CAZy genes having increased transcript abundance at 24 h, this time period would allow the substantial differences in enzyme activities measured in the monocultures to have an effect on the transcript abundance of the other fungi in the mixed cultures, for example via signalling through sugars (which could act as inducers of CAZymes) released from the straw. Furthermore, as enzymatic lignocellulose degradation can be a relatively slow process, the 24 h time-point could allow for various polysaccharide layers to be broken down perhaps exposing sugar inducers that would otherwise not be exposed at an earlier time point.

The enzymatic assays using the pNP-linked carbohydrate substrates (with the exception of the glucopyranoside which results in the release of glucose) suggested what sugars (the sugars xylose, arabinose, galactose and cellobiose can act as inducers of CAZymes) could be released at the 24 h time point in monocultures (Fig. 1). To quantify some of the actual inducers from mono and mixed cultures, the monosaccharides were quantified in the hydrolysates from saccharification reactions (performed at the same temperature and in the same media used to culture the fungi) that used enzymes concentrated from 24 h culture filtrates. As the fungus will import and metabolise the sugars hydrolysed from the straw, this approach rather than measuring the monosaccharides present in the culture filtrates is more likely to give an accurate indication of the flux of sugars in the shake-flask cultures. Furthermore, only monosaccharides were quantified because the flux of dimers such as cellobiose or longer cellodextrins (known inducers of *T. reesei CAZymes*) could not be measured as these dimers or cellodextrins would largely be degraded to monomers in the saccharification reactions.

#### 3.2.2 Saccharification assays with enzymes from mono and mixed cultures

**Fig. 2:**
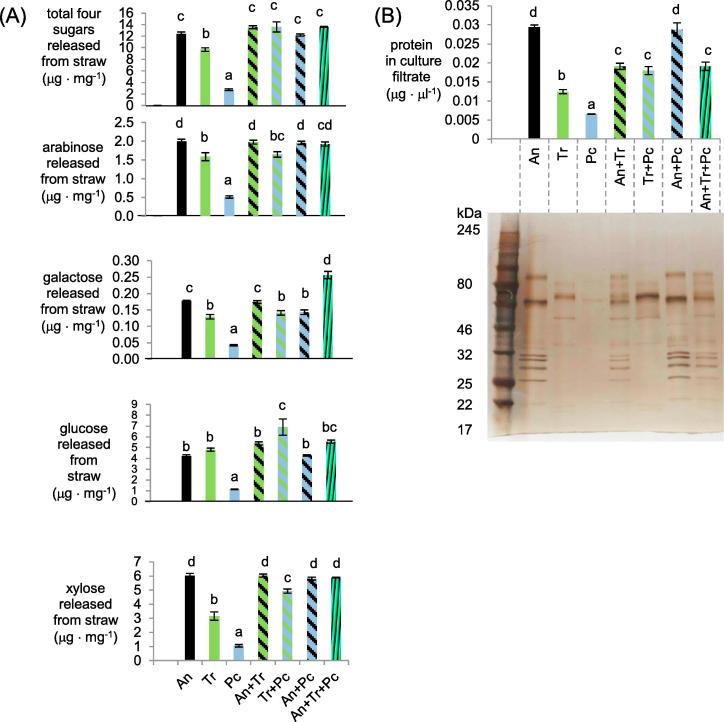
Monosaccharides (which include inducers of CAZymes) released by the fungi from straw. (A) Monosaccharides released from saccharification reactions using equivalent volumes of the cultures filtrates and (B) silver stained PAGE gel where equivalent volumes of the culture filtrate were loaded from one of the replicate set of flasks and protein concentrations in the culture filtrates. Error bars represent standard errors. Bars in a chart that contain the same letter were not significantly different (Tukey’s post-hoc test (*p* < 0.05) after ANOVA analysis).

Saccharification assays were performed (at the same temperature and in the same media used to culture the fungi) using volumes of the concentrated proteins equivalent to what would be present in the different shake-flask cultures so as to predict what would be the flux of sugars in each culture (Fig.2A). The saccharification assays identified three beneficial effects of mixed cultures with regard to sugar release. Firstly, the enzymes from the *A. niger* monoculture filtrates and mixed cultures of *T. reesei* that included *A. niger* released significantly more sugars from hemicelluloses (as measured by arabinose, galactose and xylose) than the *T. reesei* monoculture filtrates, whereas there were no significant differences in the sugar release from cellulose (as measured by glucose). Secondly, the release of glucose and xylose from enzymes from the *T. reesei* and *P. chrysogenum* mixed culture was significantly greater than the glucose and xylose released using enzymes from either of the monocultures. Thirdly, the release of galactose using the enzymes from the tri-species mixed cultures was significantly higher than the release from using the enzymes from any of the three monocultures. For all other comparisons, the amounts of monosaccharides released using the enzymes from mixed cultures were not significantly greater than what was released by the enzymes from all of the monocultures that were also part of that mixed culture. Galactose was the only sugar where there was an overall improvement i.e., where the release from straw using the enzymes from the mixed culture was higher over all of what was released by the monoculture filtrate enzymes. Each of the four monomers was detected in every saccharification reaction indicating that the sugar differences in the shake-flask cultures are in terms of concentration rather than the absence or presence of the sugar. To indicate whether these levels of activities could lead to any accumulation of sugars that could be inhibitory of CAZy transcript abundance rather than inducing, the monosaccharide concentrations in the shake-flask cultures at 24 h were also measured (Supplementary Figure S2). The monosaccharide levels were all below 100 μM and thus lower than what has been shown for *A. niger* to be inhibitory of CAZyme gene expression via CreA (de Vries et al., 1999).

#### 3.2.3 Protein concentrations from filtrates from mono and mixed cultures

To aid interpretation of the different levels of activities, the proteins from the concentrated culture filtrates were separated by PAGE gels and quantified (Fig.2B). The protein quantifications correlated well with the PAGE gel staining intensity of equivalent volumes of culture filtrates. The lowest protein concentrations were from the enzymes from the *P. chrysogenum* monocultures which also had the lowest sugar releasing enzyme activities. The protein concentration from the *T. reesei* and *P. chrysogenum* mixed culture was significantly greater than either of the respective monocultures, which could in part explain the significantly higher glucose and xylose released by the enzymes from the mixed culture. In other cases, the protein concentrations in the mixed cultures were not significantly greater than all of the monocultures that were also part of the mixed cultures. The approximately four-fold higher protein concentrations and predicted flux of the monosaccharides in the mixed cultures with *P. chrysogenum* compared to the monoculture indicated the potential for beneficial effects on *P. chrysogenum* CAZy transcript abundance. We hypothesised that *P. chrysogenum* would show more differences or at least more clearer differences in CAZy gene transcript abundance than would be the case for the other two fungi because with those fungi the protein concentrations and flux of sugars was more similar between the mono and mixed cultures. This hypothesis was one of the reasons that led us to include *P. chrysogenum* in the RNA-seq experiment. We also determined whether there would be sufficient RNA from each fungus in all the mixed cultures at the 24 h time-point.

#### 3.2.4 Measurements of total RNA from each species initially and after a period of 24 h in straw cultures

rRNA is used for identification of fungal species (White et al., 1990) as well as a reference gene for normalisation for total RNA abundance in qRT-PCR (Shi and Chiang, 2005). Therefore the rRNA region is ideally suited to quantify species-specific abundance of total RNA. With regard in particular to our study, the 5.8S region was used to (1) estimate the amount of total RNA present to guide the experimental conditions for RNA-seq so as to obtain sufficient numbers of RNA-seq reads from each fungus and (2) indicate how the fungi were interacting in the mixed cultures through changes in the relative amounts of total RNA from each fungus over time. The 5.8S region of the rRNA extracted from each culture was quantified using species-specific primer and hydrolysis probe combinations. The quantification of the 5.8S rRNA showed changes in abundance in the mixed cultures over time (Supplementary Figure S3). With regard to mixed cultures that included *T. reesei*, the quantification of the 5.8S rRNA indicated that *T. reesei* would likely be the source of the majority of the reads when present in a 24 h mixed culture but still have sufficient numbers of reads from the other fungi in these mixed cultures for two reasons. Firstly, the amounts of *A. niger* and *P. chrysogenum* 5.8S rRNA in mixed cultures with *T. reesei* were still approximately 10–20% of the levels of *T. reesei*. Secondly, different total numbers of reads can be obtained from individual samples from RNA-seq, so in this study approximately 100 M reads were sequenced from mixed culture samples compared to 30 M reads from monoculture samples. With regard to the mixed cultures of only *A. niger* and *P. chrysogenum,* there were likely to be similar numbers of reads obtained from each fungus in the mixed culture samples.

**Fig. 3:**
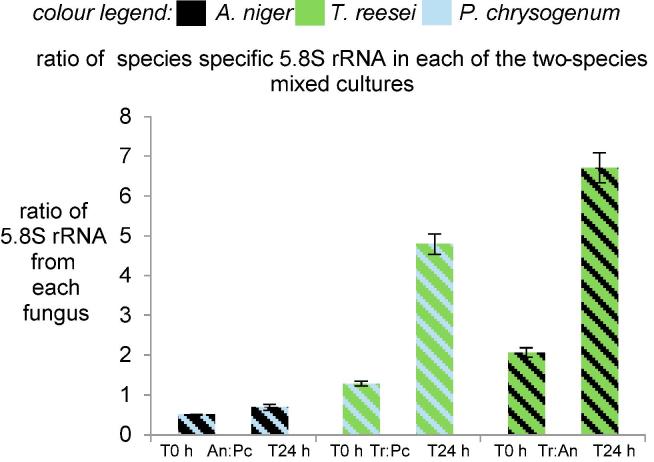
The ratio of the 5.8S rRNA from each species present in the two-species mixed cultures. The ratio was used to indicate the relative changes in total RNA in the two-species mixed cultures over 24 h. The error bars represent the standard errors from cultures from three replicate flasks.

The ratios of the species-specific 5.8S rRNA in the two species mixed cultures illustrate clearly how the amounts of 5.8S rRNA had changed in the mixed cultures over 24 h (Fig. 3). In the two species mixed culture with *A. niger* and *P. chrysogenum*, the ratio of the 5.8S rRNA only changed marginally by 24 h. In contrast, in the two species mixed cultures with *T. reesei* and *A. niger* or *T. reesei* and *P. chrysogenum*, the ratio of the *T. reesei* 5.8S rRNA to the 5.8S rRNA from the other fungus increased by approximately 4-fold. This increase in the ratio of *T. reesei* 5.8S rRNA suggested there could be either a faster growth rate of *T. reesei* or antagonism leading to a decline in the biomass of the other two fungi or combinations of both these suggestions. This was investigated by estimating the biomass in mixed cultures.

#### 3.2.5 Measurement of mycelial biomass using gDNA

We determined the mycelial dry weight of each fungus in mixed cultures via gDNA isolated from the initial mycelial biomass added to the flasks (0 h) and after 24 h in straw cultures (Supplementary Figure S4). With regard to *T. reesei*, there was a significant increase in mycelial biomass in the three species mixed culture at 24 h compared to the *T. reesei* biomass in the 24 h monoculture. The *T. reesei* mycelial biomass in mixed cultures did not decrease significantly below the biomass in the *T. reesei* monoculture at 24 h. In contrast, for *A. niger*, there was significant decreases in biomass in both of the mixed cultures with *T. reesei* compared to the *A. niger* monoculture at 24 h. In mixed cultures with only *A. niger* and *P. chrysogenum*, there was no significant change in *P. chrysogenum* mycelial biomass at 24 h compared to the 24 h monoculture whilst there was a small decrease in *A. niger* biomass (less than half the decrease that was measured in *A. niger* biomass in the mixed cultures with *T. reesei*). The smaller reduction or lack of change in mycelial biomass in the *A. niger* and *P. chrysogenum* mixed culture correlated with the lack of change in the ratio of 5.8S rRNA from each species. Thus, the increase in the proportion of *T. reesei* 5.8S rRNA in mixed cultures with either or both of *A. niger* or *P. chrysogenum* can be explained primarily by a decline in *A. niger* mycelial biomass and secondarily by some increases in *T. reesei* mycelial biomass. The decrease in mycelial biomasses of *A. niger* in mixed cultures with *T. reesei* is indicative that *T. reesei* has antagonistic effects on *A. niger*. Other studies have inferred antagonistic effects from measuring reductions in mycelial biomass in mixed cultures such as Jonkers et al. (2012) where the mycelial biomasses decreased in mixed cultures of two maize-infecting fungi with known antagonistic features. Using the mycelial dry weight predictions, the monosaccharides released from saccharification assays using the 24 h cultures filtrates were expressed as a proportion of these weights (Supplementary Figure S5). This clearly illustrated competitive or antagonistic aspects of the mixed cultures as the monosaccharides released from mixed culture filtrates never released more sugars than the monoculture filtrates when expressed per mycelial dry weight of the culture.

### 3.3 Analysis of transcript abundance in mono and mixed cultures using dual RNA-seq

#### 3.3.1 Summary of the total number of reads from each species mapped in mono and mixed cultures

RNA-seq analysis involves aligning reads to a reference genome and then counting the reads that align uniquely. In our study, the individual genome sequences from each species were concatenated so as to create a tri-species reference genome so then any reads that were not unique amongst the three species would not be counted. The number of reads that mapped uniquely to each species from each sample is summarised in Supplementary Table S4. Sufficient sequencing depth (between 7 M and 100 M reads) was obtained from each species from mixed cultures samples for statistical analysis comparing transcript abundance amongst the glucose monoculture conditions and mono and mixed species straw culture conditions.

There was a positive correlation as measured by the squared Pearson correlation coefficient (r^2^) of 0.74 between the copy number of the 5.8S rRNA region from each species from a mixed culture sample and the number of uniquely mapped reads obtained from each species from the same sample (Supplementary Figure S6). This finding supports the utility of the 5.8S rRNA quantification approach to predict the relative number of reads that would be obtained by dual RNA-seq.

FPKM is a standard normalised measure of transcript abundance from RNA-seq experiments (Mortazavi et al., 2008). In our study, the FPKM values for a particular species are normalised to the total number of uniquely mapped reads from that species instead of the total number of reads from an RNA sample. The trends in transcript abundance as measured by these FPKM values were validated by qRT-PCR for two protein encoding genes from each species (Supplementary Figure S7). For each of these genes there was a positive correlation between the FPKM values and the fold changes from qRT-PCR with the measured squared Pearson correlation coefficients (r^2^) values between 0.94 and 0.71.

#### 3.3.2 Summary of the number of differentially expressed genes in comparisons

**Fig. 4:**
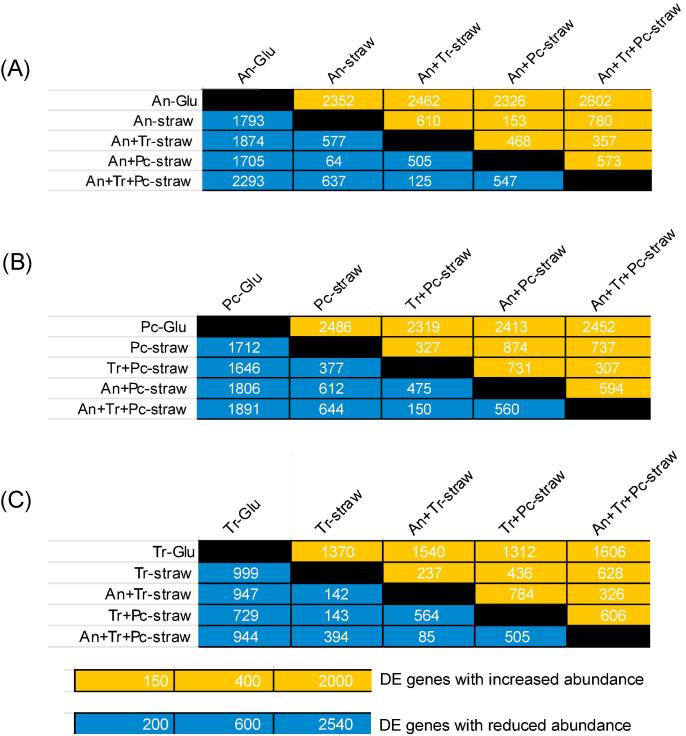
Summary of the numbers of differentially expressed (DE) genes in comparisons illustrated for (A) *A. niger*, (B) *P. chrysogenum* and (C) *T. reesei*. The control condition is listed in the rows for the genes with increased transcript abundance and in the columns for the genes with reduced transcript abundance. The differentially expressed genes have an FPKM ⩾ 1 in the test condition for the genes with increased transcript abundance and in the control condition for genes with reduced transcript abundance as well as ⩽⩾2-fold change in expression (DESeq2 counts) and *p*_adj_ < 0.05.

The number of differentially expressed (DE) genes varied in the comparisons of transcript abundance in the mono and mixed cultures (Fig. 4). For each of the three species, there were more DE genes when straw cultures were compared to the respective glucose controls than when mono and mixed species straw cultures were compared to each other. More *A. niger* genes were differentially expressed in comparisons of the *A. niger* straw monocultures with mixed cultures that included *T. reesei* than those that only included *P. chrysogenum* (Fig.4A). The smallest number of total DE genes was in the comparison of the *A. niger* straw monoculture to the *A. niger* and *P. chrysogenum* mixed culture where there were only 153 genes with increased transcript abundance and 64 genes with reduced transcript abundance (Fig.4A), indicating that co-cultivation with *P. chrysogenum* hardly affects transcript abundance in *A. niger*. On the contrary, *P. chrysogenum* is affected strongly by co-cultivation with *A. niger* as there were more DE genes (with either increased or decreased abundance) in the mixed cultures that included *A. niger* than those that only included *T. reesei* (Fig.4B). With regard to *T. reesei* DE genes, there were more genes with increased abundance in mixed cultures that included *P. chrysogenum* than those that only included *A. niger* when compared with the *T. reesei* straw monoculture (Fig.4C). The number of *T. reesei* genes with reduced transcript abundance in mixed cultures with either *A. niger* or *P. chrysogenum* compared to the *T. reesei* straw monoculture was similar.

#### 3.3.3 GO term enrichment analysis of the differentially expressed genes

**Fig. 5:**
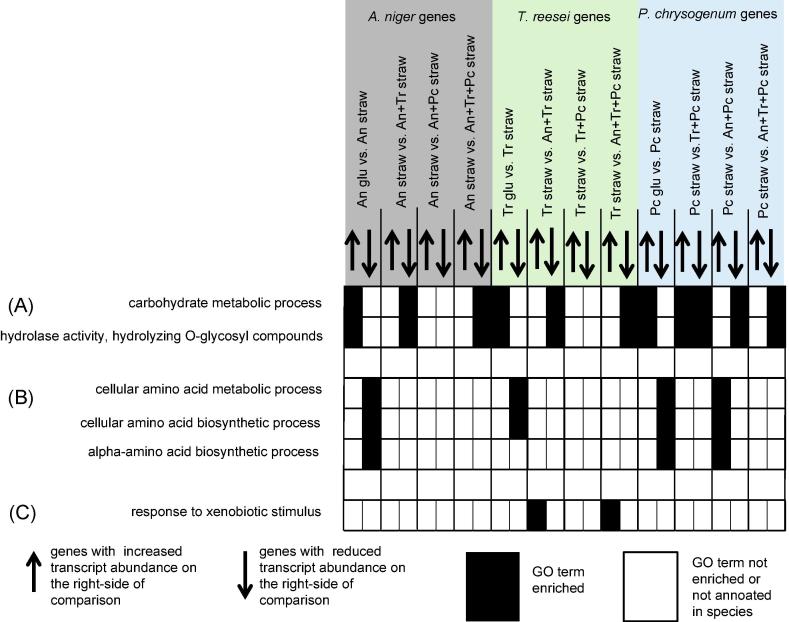
Summary of the enrichment pattern of selected GO terms in DE genes from the straw monoculture compared with either the respective glucose control or any mixed species cultures that also contained that species. An = *A. niger*, Tr = *T. reesei* and Pc = *P. chrysogenum.* See Supplementary Table S5 for a list of all the enriched GO terms as well as the lists of genes responsible for the enrichment of a GO term.

Gene Ontology enrichment analysis was performed using GOEAST to provide an overview of the trends in the DE genes (Fig. 5 and Supplementary Table S5). The DE genes analysed here were those of the straw monocultures compared with either the respective glucose control or any mixed species cultures that also contained that species. GO terms related to the breakdown of complex carbohydrates are commonly found in transcriptomic analyses of fungi when transferred from simple carbon sources to lignocellulose. In this study, the GO terms ‘carbohydrate metabolic process’ and ‘hydrolase activity, hydrolysing O-glycosyl compounds’ were enriched in genes with increased abundance in the straw monocultures compared to the respective glucose controls (Fig.5A). These GO terms were enriched in the genes with reduced transcript abundance in seven of the nine comparisons that compared a straw monoculture to a mixed culture that also contained that species (Fig.5A). The *P. chrysogenum* genes with increased transcript abundance in the mixed cultures with *T. reesei* were enriched for the GO terms related to the breakdown of complex carbohydrates. This was the only group of genes with increased transcript abundance in mixed cultures compared to the straw monoculture that were enriched for these GO terms. Apart from polysaccharide breakdown GO terms, there was an enrichment pattern for GO terms related to amino acid biosynthesis that suggested that *P. chrysogenum* in mixed cultures with *A. niger* was synthesising more amino acids than in the straw monoculture (Fig.5B). The GO term for ‘cellular amino acid biosynthetic process’ was enriched in the *P. chrysogenum* genes with increased abundance in the mixed culture with *A. niger* as well as in the genes with reduced abundance in the *P. chrysogeum* straw monoculture compared to the glucose control. The GO term for ‘response to xenobiotic stimulus’ was enriched in the *T. reesei* genes with increased abundance in mixed cultures with *A. niger* compared to the *T. reesei* straw monoculture (Fig.5C). The *T. reesei* genes annotated with this GO term responsible for the enrichment have the Pfam domain for RTA1-like protein (PF04479) and these membrane-bound proteins can have a detoxifying role. One study showed that supression of an RTA1-encoding gene led to lower ethanol production from a straw/bran mix by *Fusarium oxysporum* (Ali et al., 2014). These *T. reesei* genes could be induced here to detoxify components of the lignocellulose released in the mixed culture or produced by *A. niger*. Production of compounds toxic to a competing fungus is a prominant feature of fungal interaction (Boddy, 2000, Jonkers et al., 2012).

### 3.4 CAZy transcript abundance in the mono and mixed cultures

#### 3.4.1 Overview of CAZy abundance changes in mixed cultures

**Fig. 6:**
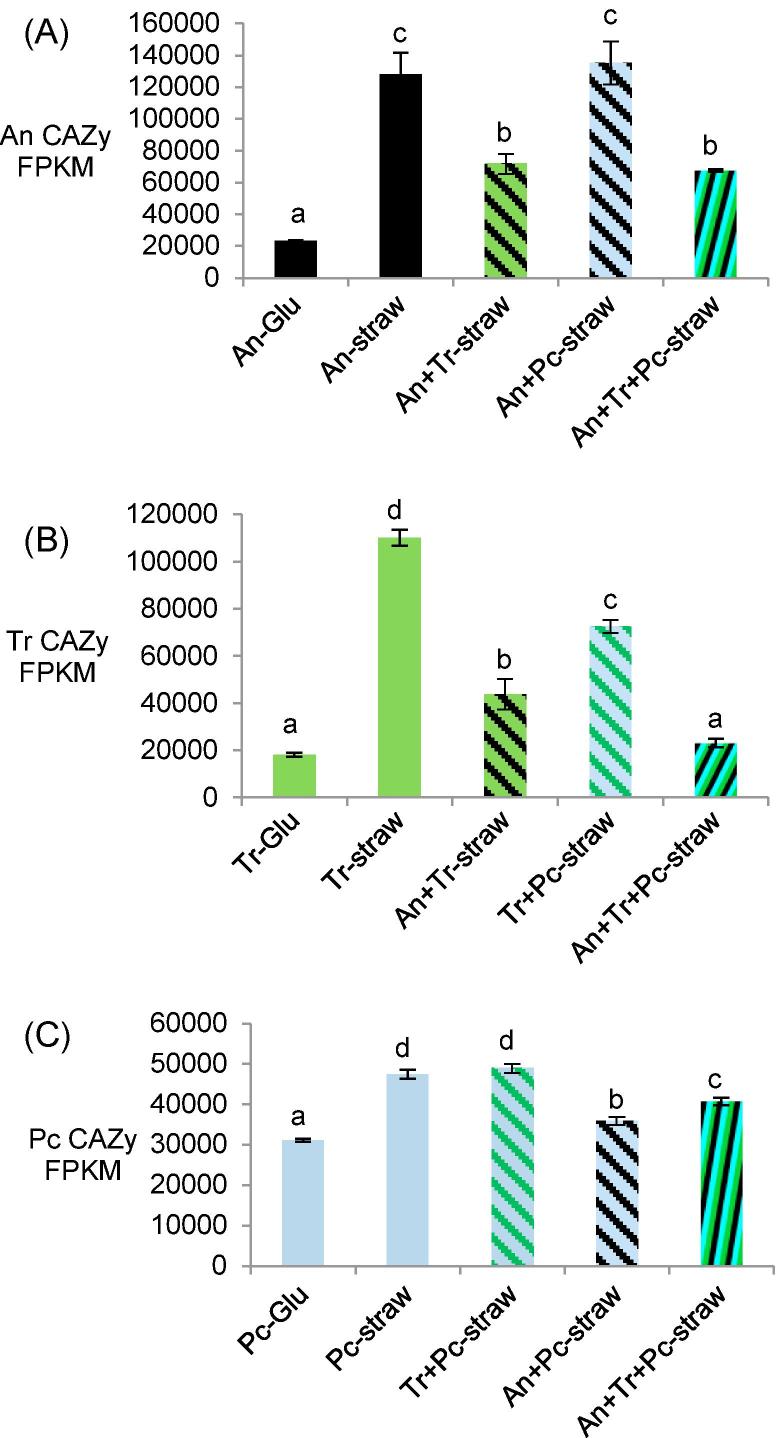
Total CAZy transcript abundance in glucose and straw monocultures and mixed species straw cultures for (A) *A. niger*, (B) *T. reesei* and (C) *P. chrysogenum*. Bars in the chart that contain the same letter were not significantly different (Tukey’s post-hoc test (*p* < 0.05) after ANOVA analysis). Error bars represent standard errors (n = 3).

The total transcript abundance attributed to CAZy genes (excluding glycosyltransferases (GTs) as these are not involved in the degradation of lignocellulose) was calculated (Fig. 6). For each of the three species, the total numbers of CAZy transcripts were higher in the straw monocultures compared to the respective glucose controls. There were differences for each of the species in how the total CAZy transcript abundances in the mixed cultures compared to the respective straw monocultures. With regard to *A. niger* total CAZy transcript abundance, this was significantly lower in the mixed cultures that included *T. reesei* but was not significantly different in the mixed culture with only *P. chrysogenum* compared to the *A. niger* straw monoculture (Fig.6A), confirming the earlier observation that *A. niger* transcript abundance was not affected much by mixed cultivation with *P. chrysogenum*. *T. reesei* total CAZy transcript abundance was significantly lower in both of the two species mixed cultures and lower still in the three species mixed culture than in either of the two species mixed cultures (Fig.6B). The *P. chrysogenum* total CAZy transcript abundance was not significantly different in the mixed culture with only *T. reesei,* whereas the levels were significantly lower in the mixed culture with only *A. niger* as well as the three species mixed culture (Fig.6C). These differences in total CAZy transcript abundance in mixed cultures indicated contrasting interactions between the fungi. Notably, none of the mixed cultures led to higher total CAZy transcript abundance than the respective straw monocultures.

Relatively few CAZy genes had increased abundance in mixed cultures but not in the straw monocultures (23/200 *A. niger* CAZy genes, 18/136 *T. reesei* CAZy genes and 10/139 *P. chrysogenum* CAZy genes) (see Venn diagrams in Supplementary Table S6). Genes encoding fungal cell wall-acting enzymes were found to be induced in subsets of the straw cultures and these are described in more detail in a later section. With regard to genes encoding plant biomass degrading CAZymes, one example is the *T. reesei* α-l-arabinofuranosidase (Trire2_55319 – *abf3*) which had increased transcript abundance in mixed cultures that included *A. niger* but not in mixed cultures with only *P. chrysogenum* or in the *T. reesei* straw monoculture.

#### 3.4.2 Subset of *P. chrysogenum* CAZy genes having higher transcript abundance in mixed cultures with *T. reesei*

**Fig. 7:**
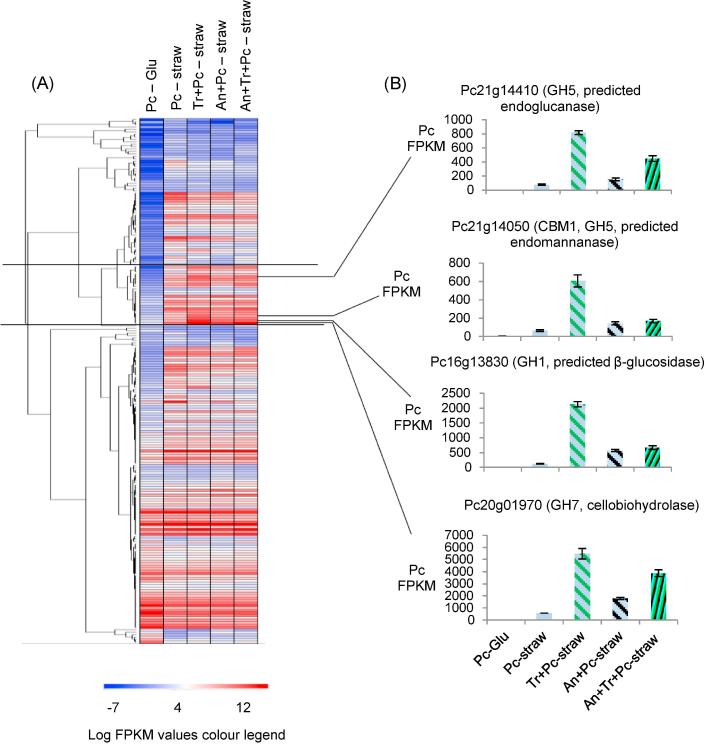
(A) Hierarchical clustering of the *P. chrysogenum* CAZy genes and (B) selected examples of *P. chrysogenum* CAZy genes that have higher abundance in the mixed culture with *T. reesei* and to a lesser extent in other mixed cultures. Error bars represent standard errors (n = 3). See Supplementary Figure S8 for a high resolution image of the clustering heatmap where the gene that each row represents in labelled.

**Supplementary Figure S8:**
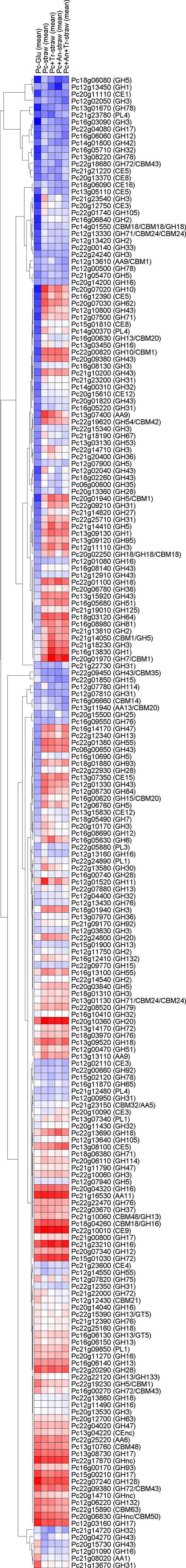
High resolution image of the hierarchical clustering of the *P. chrysogenum* CAZy genes from Fig.7(A).

The FPKMs of *P. chrysogenum* CAZy genes from mono and mixed cultures were clustered. There was a cluster of 24 *P. chrysogenum* CAZy genes (highlighted cluster in Fig.7A) where the predominant trend was higher transcript abundance in the mixed cultures compared to the *P. chrysogenum* straw monoculture. This cluster included 14 genes that had significantly (*p*_adj_ < 0.05) increased transcript abundance in the two species mixed culture with *T. reesei* compared to the *P. chrysogenum* straw monoculture. These genes included those encoding for a cellobiohydrolase, endo-glucanase, endo-mannanase, β-mannosidase and two intracellular β-glucosidases (Fig.7B and Supplementary Figure S8). Their transcript abundance profile could be due to the greater flux of sugars (xylose, arabinose and galactose could act as inducers of CAZymes) in this mixed culture, as the saccharification assays showed that there was five-fold more total monosaccharides released using the *T. reesei* and *P. chrysogenum* 24 h mixed culture filtrate compared to the *P. chrysogenum* 24 h monoculture filtrate (Fig.2A). There was also increased transcript abundance for some of the genes in this cluster in the mixed culture with *A. niger* and the tri-species mixed culture but generally to a lesser extent than in the mixed culture with *T. reesei*. The orthologue of the *P. chrysogenum* cellobiohydrolase (*Pc20g01970*) from the above cluster in a cold-adaptive *P. chrysogenum* strain was shown to be induced by several sugars (sophorose, cellobiose, gentiobiose, lactose and xylose) at a 1% concentration (Hou et al., 2007). There were other *P. chrysogenum* CAZy genes that had reduced transcript abundance in the two species mixed culture with *T. reesei* as indicated by the lower intensity colours in the heatmap. The increased transcript abundance of this small subset of *P. chrysogenum* CAZy genes in the mixed cultures demonstrates a beneficial effect of mixed cultures on CAZy transcript abundance although the total *P. chrysogenum* CAZy transcript abundance in any of the mixed cultures is not higher than the *P. chrysogenum* straw monoculture (Fig. 6).

#### 3.4.3 Subset of *T. reesei* CAZy genes with reduced transcript abundance in mixed cultures

**Fig. 8:**
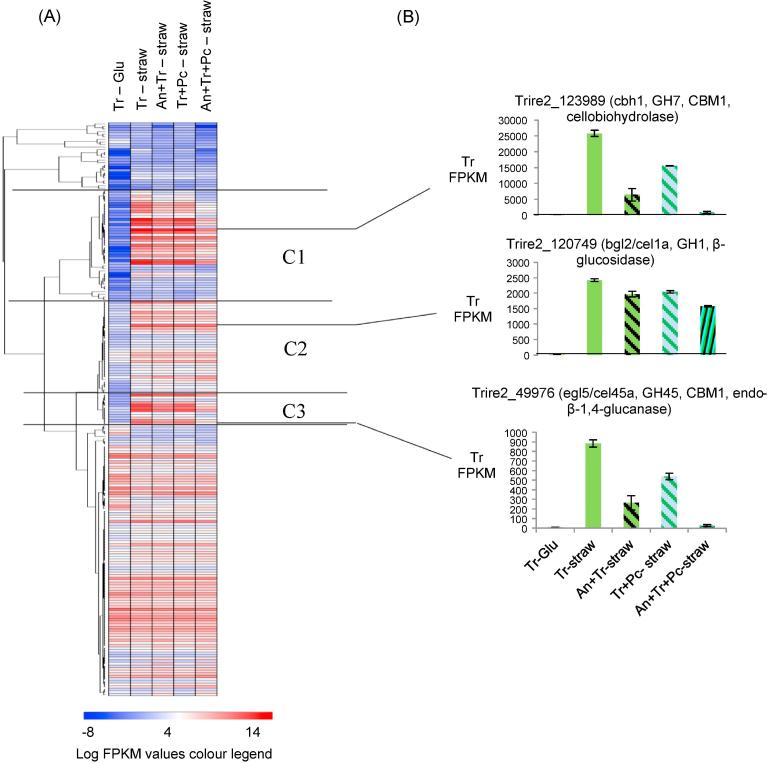
(A) Hierarchical clustering of the *T. reesei* CAZy genes and (B) selected examples of *T. reesei* CAZy genes that are members of the indicated clusters. Error bars represent standard errors (n = 3). See Supplementary Figure S9 for a high resolution image of the clustering heatmap where the gene that each row represents in labelled.

The FPKM values of *T. reesei* CAZy genes from mono and mixed cultures were clustered. One prominent trend was a decrease in the transcript abundance of *T. reesei* CAZy genes in mixed cultures compared to the *T. reesei* straw monoculture (Fig.8A). Two clusters with this trend, C1 and C3 (Fig.8A), contained 55 genes that included many of the genes that encode the major CAZymes involved in plant biomass degradation (Supplementary Table S2). There was a large overlap between the genes in these clusters that were significantly lower in the two species mixed cultures with the genes that were significantly lower in the tri-species mixed culture. All of the 38 genes with significantly (*p*_adj_ < 0.05) lower transcript abundance in the *A. niger* and *T. reesei* two species mixed culture and 34/36 of the genes with significantly (*p*_adj_ < 0.05) lower transcript abundance in the *T. reesei* and *P. chrysogenum* two species mixed culture were amongst the 44 genes with significantly (*p*_adj_ < 0.05) lower abundance in the tri-species mixed culture. The mean fold change reduction in transcript abundance was larger in the tri-species mixed culture (10-fold) than in the two species mixed culture of *T. reesei* with *A. niger* (3-fold) or with *P. chrysogenum* (1.8-fold). There were other *T. reesei* CAZy genes that generally did not have lower transcript abundance in the mixed cultures and these mainly clustered in C2. The enzyme activities or total protein levels in the 24 h mixed cultures with *T. reesei* were not synergistically greater than in the *T. reesei* straw monocultures (Fig. 2), which could be explained by the reduced transcript abundance of the *T. reesei* CAZy genes. One could speculate on factors that could contribute to this reduced transcript abundance in mixed cultures; there could be higher rates of uptake of sugars (some of which are inducers of CAZymes) by *A. niger* and *P. chrysogenum* transporters compared to *T. reesei*, thus restricting access of *T. reesei* to these sugars. Competitive and antagonistic factors are likely contributors also where *T. reesei* could be diverting its limited resources to respond to aggression from the other fungi or antagonising the other fungi.

### 3.5 Saccharification level from 4 d mixed cultures showed limited improvements compared to monocultures

**Fig. 9:**
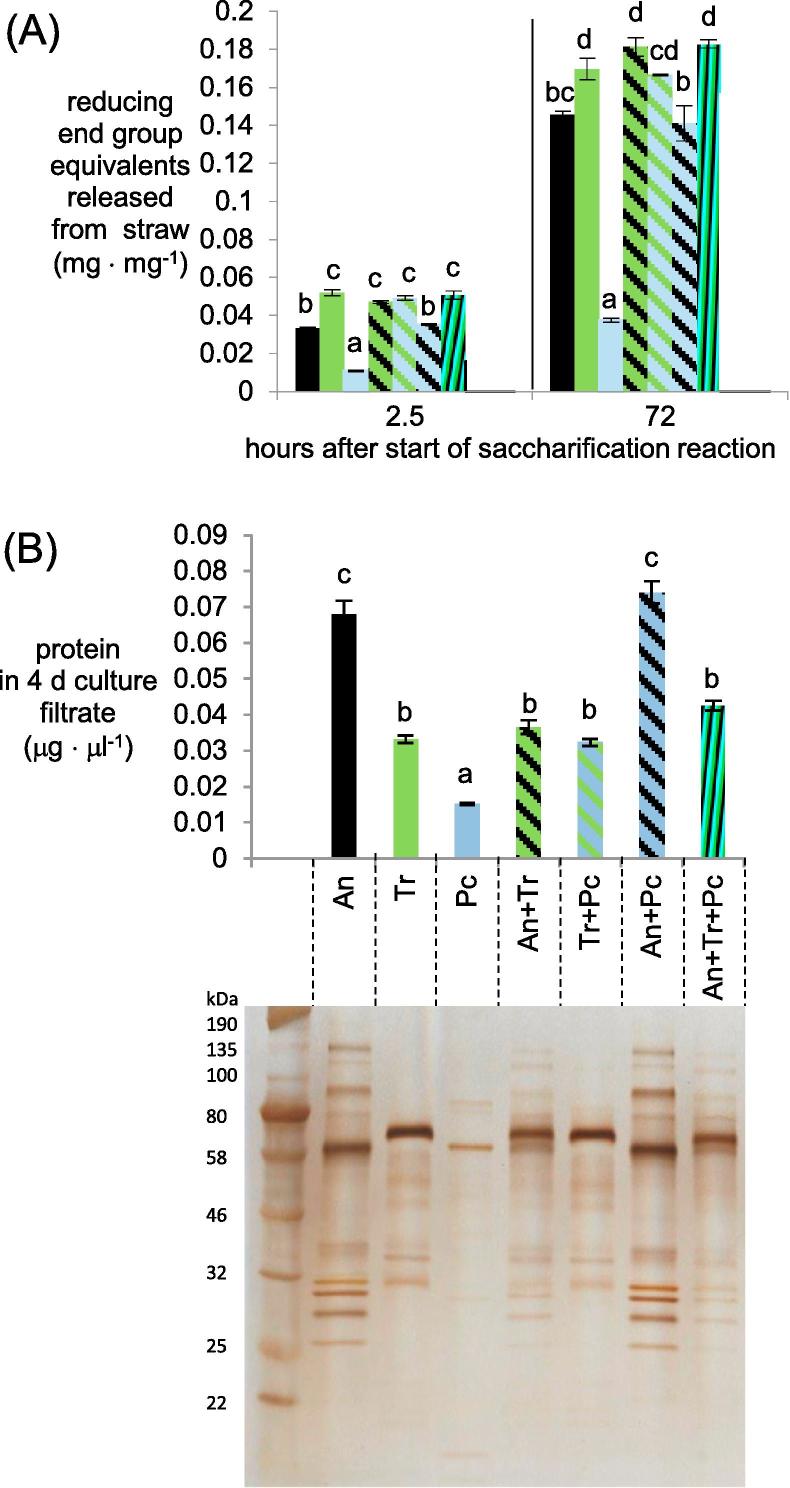
Saccharification using 4 d culture filtrates. (A) Saccharification of wheat straw in standard-type saccharification assays where equivalent volumes of the filtrates were used from 4 d cultures and (B) protein quantities from the 4 d culture filtrates and silver stained PAGE gel where equivalent volumes of the culture filtrate were loaded from one of the replicate set of flasks. Bars in the chart that contain the same letter were not significantly different (Tukey’s post-hoc test (*p* < 0.05) after ANOVA analysis). Note: that the statistical analysis on each of the saccharification time points was done separately. Error bars represent standard errors.

We tried to predict whether the mixed cultures saccharified the wheat straw in the shake-flask cultures to a greater level compared to monocultures and whether the mixture could have industrial applications. The enzymatic activities from culture filtrates from mono and mixed cultures 4 d after transfer were assayed in standard-type saccharification assays (incubated at 50 °C in citrate buffer pH 4.8) (Fig.9A). A later time point than was used for the RNA-seq was chosen here in order to allow a substantial amount of enzymes to accumulate from the *P. chrysogenum* monoculture (where secretion of proteins appeared to be lower compared to the other fungi). The saccharification assays were performed using equivalent shake-flask volumes of the concentrated culture filtrate from each culture and aliquots were taken to measure reducing end groups at various time points. The reducing end groups released using enzymes from filtrates of mixed cultures that included *P. chrysogenum* were significantly higher than those released using enzymes from *P. chrysogenum* monocultures. Otherwise, the reducing end groups released from the straw from the mixed culture filtrates were not significantly greater than what was released from all of the monoculture filtrates of the fungi that were included in the mixed culture (Fig.9A). The same trend was reflected in the protein concentration from the filtrates from the 4 d cultures where the protein concentrations were only significantly higher in mixed cultures with *P. chrysogenum* compared to the *P. chrysogenum* monoculture, but otherwise mixed cultures did not have significantly higher protein concentrations than the monocultures that composed the mixed culture (Fig.9B). *T. reesei* became more dominant in the mixed cultures but these cultures still contained the other fungi as their gDNA was detected. The mycelial dry weight prediction is not shown because gDNA from 4 d cultures was partly degraded which can affect the accuracy of the prediction. Also, the banding pattern from the PAGE gel indicated that in the mixed cultures involving *A. niger* and *T. reesei*, the proteins present were a mixture of those secreted by each fungus (Fig.9B). The detrimental effects of *T. reesei* CAZy transcript abundance in mixed cultures could explain why there was not an improvement in saccharification from the *T. reesei* mixed cultures.

### 3.6 Competitive or antagonistic interactions in the mixed cultures

When fungi interact, competitive interactions are commonplace (Boddy, 2000) and the limited increase in CAZy transcript abundance and enzyme activities in mixed cultures could be explained by competitive or antagonistic interactions between the fungi suggested previously by the decline in mycelial biomasses in mixed cultures as well as enrichment of GO terms that could be related to detoxifying compounds in mixed cultures. The genes that were induced most highly in the mixed cultures compared to the straw monocultures were examined for evidence of competitive interactions (Supplementary Table S7).

There were genes from each fungus that were highly induced in the mixed cultures with each of the other fungi which indicated that antagonistic interactions were occurring in the mixed cultures (Supplementary Table S7). Oxidoreductases can have a role in response to fungal antagonism such as cytochrome P450 oxidoreductases that function in synthesising or detoxifying toxic compounds (Črešnar and Petrič, 2011). Genes from each of the three species encoding oxidoreductases were commonplace amongst the highly induced genes in the mixed cultures. This is in agreement with two studies from the literature where the majority of the most highly induced genes in a basidomycete interaction were also encoding oxidoreductases (Arfi et al., 2013) and the higher expression of genes encoding oxidoreductases was part of the response of two plant infecting fungi in their interaction (Jonkers et al., 2012). 11/18 of the *A. niger* oxidoreductases that were highly induced in mixed cultures are part of secondary metabolite clusters and these are analysed in more detail in a later section using the annotation of Inglis et al. (2013). To our knowledge no similarly extensive annotation of secondary metabolite clusters is available for *T. reesei* or *P. chrysogenum* other than for the backbone synthesising enzymes annotated by Martinez et al. (2008) or van den Berg et al. (2008). An analysis of the genomic locations of the highly induced *T. reesei* or *P. chrysogenum* oxidoreductases in mixed cultures shows that these genes are not generally located within 15 gene models of the backbone synthesising enzymes indicating they are likely not located in any secondary metabolite clusters (with the exceptions of *Trire2_105874* near the PKS-NRPS *Trire2_105864, Pc21g04770* near the PKS *Pc21g04840* and *Pc21g12590* near the NRPS *Pc21g12630*).

Glutathione S-transferases (GSTs) are ubiquitous enzymes that function in the conjugation of toxic compounds thereby reducing the toxicity (Veal et al., 2002). Two different *T. reesei* genes encoding GSTs were highly induced in the tri-species mixed culture and in the two species mixed culture with *P. chrysogenum* (Supplementary Table S7) which may be functioning in these mixed cultures to detoxify compounds synthesised by the other fungi. Arfi et al. (2013) found six GSTs amongst the most highly induced genes in a basidiomycete interaction. Transporters can function in removal of toxic compounds from a cell including toxic compound made during fungal interaction. Genes encoding ABC transporters and drug transporters were highly induced in each of the three species in their interactions with the other fungi. In particular, an *A. niger* drug transporter was highly induced in the mixed cultures that included *T. reesei* as well as *T. reesei* drug transporters in mixed cultures that included *P. chrysogenum* (Supplementary Table S7). ABC efflux transporters and drug resistance transporters were induced in mixed cultures involving *Trichoderma* species and a plant pathogenic fungus (Atanasova et al., 2013) and drug transporters were induced in plant infecting fungi when they interacted (Jonkers et al., 2012).

Analysis of the highly induced genes in the mixed cultures provided evidence for competitive or antagonistic interactions. To further understand the fungal biology of these interactions in the mixed cultures, we investigated using the RNA-seq data known methods of antagonism (such as expression of fungal cell wall hydrolyases or secondary metabolite cluster gene expression) or consequences of antagonism (stresses such as starvation and sporulation).

### 3.7 Fungal interaction in mixed cultures as indicated by expression of fungal cell wall modifying hydrolases

Fungal cells are surrounded by cell walls that are important during contact with the environment, including interactions with other species. The cell wall is dynamic and changes composition in response to fungal development and environmental factors. It consists of a carbohydrate network composed of β-1,3-glucan, chitin and species-specific polymers such as α-1,3-glucan, galactomannan and galactosaminogalactan (Latge and Beauvais, 2014). We analysed expression of genes from CAZy families that are reported to be involved in fungal cell wall synthesis, modification and degradation to indicate whether the fungi were growing, starving and/or sporulating in mixed cultures as well as whether the genes encoding fungal cell wall degrading enzymes could be antagonistic factors by degrading the cell walls of another fungus.

#### 3.7.1 Growth and starvation as indicated by fungal cell wall modifying enzymes

The *A. niger* transcript abundance patterns for fungal cell wall CAZy indicated adversely affected growth and increased carbon starvation in all straw cultures but most pronounced in those cultures with *T. reesei,* which concurs with the observed decrease in *A. niger* biomass in mixed cultures with *T. reesei* compared to monocultures (Supplementary Figure S4). Looking in detail at the expression patterns in *A. niger* that led to these conclusions; genes expressed to high levels in glucose conditions included those encoding β-glucanase BgtE and cell wall-anchored chitinase CtcA, the orthologue of hyphal tip and branch-site localised *A. nidulans* ChiA (Yamazaki et al., 2008), which are involved in modifying cell walls during hyphal tip extension (Supplementary Table S8). Transfer to straw reduced transcript levels of these growth related hydrolases ∼2-fold in the monoculture and two species mixed culture with *P. chrysogenum,* and a further 3-fold in the two species mixed culture with *T. reesei*, suggesting that growth of *A. niger* is adversely affected on straw and more so in mixed cultures with *T. reesei*. In all *A. niger* cultures on straw, genes were induced that encode the carbon starvation responsive GH47 Aspni7_PID_1156809 as well as chitinase ChiB/CfcA and GH81 β-glucanase EngA, which have a function in carbon starvation linked fungal cell wall degradation (autolysis) (van Munster et al., 2015a, van Munster et al., 2015b, Yamazaki et al., 2007). Expression levels were highest in mixed cultures with *T. reesei*. Only co-cultivation of *A. niger* with *T. reesei* on straw resulted in high induction of carbon starvation responsive GH71 alpha-1,3-glucanase AgnB and the CBM50 protein encoding *Aspni7_TID_1141444*. Reduced transcript levels for growth- and increased transcript levels for starvation related hydrolases were found for *P. chrysogenum* straw cultures, but amongst the straw cultures, the highest transcript levels for growth-related and lowest levels for starvation related genes were in the two species mixed culture with *A. niger*. This is in agreement with the enrichment of GO-terms for amino-acid biosynthesis in this mixed culture (Fig.5B). *T. reesei chiA* orthologue *Trire2_66041* and *bgtE* orthologue *Trire2_49193* had higher transcript levels in *T. reesei* monocultures and mixed cultures with *P. chrysogenum* than in mixed cultures with *A. niger*, although biomass was found to be similar in all *T. reesei* cultures. No change in expression of the starvation-linked cell wall hydrolase was found for *T. reesei* in any condition.

#### 3.7.2 Sporulation related gene expression in straw cultures

Fungi sporulate in conditions of stress and the expression pattern of genes involved in sporulation was examined in the cultures. Strong indications were found for activation of sporulation for all three species in all of the wheat straw cultures. Most of the *A. niger* sporulation related genes showed a peak in expression in the *A. niger* and *T. reesei* two species mixed culture indicating that effects of *T. reesei* in the mixed culture could be leading to greater levels of sporulation in *A. niger.* In other species, it was not clear from the expression patterns as to whether the levels of sporulation in the mixed cultures would be any higher than they already likely were in the straw monocultures. Looking in more detail at the genes and expression patterns that led to these conclusions; in *A. niger* and *P. chrysogenum*, sporulation-specific transcriptional activator BrlA and downstream regulators AbaA and WetA control expression of spore-coating hydrophobins and enzymes that remodel the fungal cell wall during sporulation (Krijgsheld et al., 2013, Prade and Timberlake, 1994). In *Trichoderma*, *brlA* is absent (Carreras-Villasenor et al., 2012). In *A. niger brlA* showed increased transcript levels in all wheat straw cultures, as did genes for spore pigment biosynthesis (*fwnA*, *brnA*, *olvA*) hydrophobins (*hyp1*, *hypB*, *hypC*, *rodA)* and sporulation-specific cell wall enzymes (*gelG*, *CBM14*, *cfcI*, *dfgA*, *bgtD*) with for most a peak in expression in the *A. niger* and *T. reesei* two species mixed culture (Supplementary Table S8). In *P. chrysogenum*, sporulation-associated genes had increased transcript levels either in all wheat straw cultures (*brlA*, *gelG* and pigment biosynthesis genes *yA*, *arpA* and *arpB*) or only in the straw monoculture (*abrA/brnA, rodA, hypA*). In *T. reesei*, *abaA* induction was observed in all wheat straw cultures, as well as increased transcription of sporulation-induced (Metz et al., 2011) cell wall enzymes.

#### 3.7.3 Mixed-culture specific increased transcript levels do not include *T. reesei* orthologues of mycoparasitic chitinases

Trichoderma species other than *T. reesei* are known for their mycoparasitism capacities, and although mycoparasitism strategy seems to be species-specific (Atanasova et al., 2013), involvement of multi-domain chitinases has been suggested and an expansion of CBM50 and CBM18 containing chitinases was observed in mycoparasitic species (Gruber et al., 2011). In *T. reesei*, genes encoding these enzymes are hardly expressed during co-cultivations, suggesting no direct antagonism is taking place, which correlates with a loss of mycoparasitism strategy in the *T. reesei* lineage (Kubicek et al., 2011). Although those particular *T. reesei* chitinases were hardly expressed, other fungal cell wall active enzyme encoding genes represented around half of the CAZy genes that had increased transcript levels (generally only around ∼2-fold) in straw mixed cultures but not in straw monocultures (9/23 *A. niger* genes, 8/18 *T. reesei* genes and 4/10 *P. chrysogenum* genes, Supplementary Table S6 and Supplementary Table S8). Whether these mixed culture induced fungal cell wall active enzymes could be antagonistic factors (by hydrolysing the cell walls of the other fungus in preference to their own) would require further experimental work. For *A. niger*, it is noteworthy that most of these genes are chitinases and/or contain chitin-binding modules. For *P. chrysogenum*, α-1,3-glucan synthesis related genes were induced in co-cultivation with *T. reesei*. *T. reesei* expressed putative α-1,3-glucanase encoding *Trire2_71532*, and two GH47 enzyme-encoding genes highest in cultures with *A. niger,* and putative N-acetyl-β-hexosaminidase encoding *Trire2_21725 highest* in cultures with *P. chrysogenum.*

### 3.8 Secondary metabolism gene transcript abundance patterns

Mixed cultivation is an exciting opportunity to investigate activation of secondary metabolite gene clusters (Bertrand et al., 2014). Fungal secondary metabolites can be activated in mixed cultures where they are used as antagonistic factors in the competition with other fungi. These metabolites are of high importance as they include toxins as well as beneficial compounds such as antibiotics and the cholesterol-reducing lovastatin. The backbones of secondary metabolites are mainly produced by polyketide synthases (PKSs) or non-ribosomal peptide synthases (NRPSs), with accessory enzymes such as oxidoreductases further modifying the structures. Their encoding genes are located in gene clusters, which are often silent under laboratory conditions where fungi are mostly cultured as monocultures (Brakhage, 2013, Keller et al., 2005). We investigated whether the interaction in our mixed cultures activated secondary metabolite clusters by examining the expression patterns of the genes encoding the metabolite backbone synthesising enzymes.

In *T. reesei*, four out of 26 PKSs, NRPSs (Martinez et al., 2008) and terpene synthases (Mukherjee et al., 2012) encoded in the genome were induced (⩾2-fold increase in expression, *p_adj_* < 0.05, expressed to 1 FPKM in at least one condition) in *T. reesei* straw monocultures compared to glucose (Supplementary Table S9). Only *Trire2_60751* was induced in mixed cultures compared to *T. reesei* straw monocultures (Supplementary Table S9), but only to a low level, indicating that *T. reesei* likely did not generate significant levels of secondary metabolites via new metabolite backbone synthesis in response to our co-cultivation conditions. However the high induction of *T. reesei* genes encoding oxidoreductases (which were not located in secondary metabolite clusters) in mixed cultures suggests that *T. reesei* may be modifying already synthesised metabolites or detoxifying metabolites produced by the other fungi (Supplementary Table S7).

The genome of *P. chrysogenum* contains 51 backbone genes (van den Berg et al., 2008), of which 3 genes were induced in at least one of the mixed cultures compared to the straw monocultures (Supplementary Table S9). The PKS encoding *Pc21g05070* had the highest increase in transcription in the tri-species mixed culture compared to the other mixed cultures, and is an orthologue to *A. niger Aspni7_TID_1080365* (orthogroup OG000226), which also had higher transcript levels in the tri-species mixed culture compared to the straw monoculture and other mixed cultures. This cluster contains multiple genes with homologs of those in the *A. nidulans* asperfuranone biosynthesis cluster. The product of this *A. nidulans* cluster was only previously characterised after cluster activation by promoter-replacement (Chiang et al., 2009), here we have identified a native expression condition for the orthologous *A. niger* and *P. chrysogenum* clusters. Asperfuranone from *A. nidulans* has *anti*-proliferative properties towards cancerous cells (Wang et al., 2010) but to our knowledge, there is no reported antagonistic function towards fungi.

The *A. niger* genome contains 79 metabolite backbone synthesising enzymes of secondary metabolite gene clusters (Inglis et al., 2013), of which 20 were induced in the *A. niger* straw monocultures compared to glucose. In mixed cultivations, 12 genes encoding the backbone enzymes were induced compared to the *A. niger* monocultures (Supplementary Table S9), of which five in all mixed cultures, and seven only in the two species mixed culture with *T. reesei*. We identified a native expression condition for the cluster *Aspni7_TID_1095536 – Aspni7_TID_1188049* (with the PKS encoded by *Aspni7_TID_1112443*) which contains genes with high homology to the *A. nidulans* asperthecin biosynthesis cluster. This product was characterised only after overproduction brought about by genetic modification (Szewczyk et al., 2008). The NRPS encoding *Aspni7_TID_1080226* has high transcript levels in co-cultivations, especially those with *T. reesei.* Its product is so far uncharacterised, but the cluster is located directly next to a cluster that shares its expression profile and has homology to the *A. nidulans* asperfuranone biosynthesis cluster (Chiang et al., 2009). The clusters encoding backbone enzymes PKS Aspni7_TID_1170931 and NRPS Aspni7_TID_1116749, which are adjacent, stand out through their high expression levels specifically in mixed cultures. These uncharacterised clusters are strong candidates for follow-up research to identify potentially new secondary metabolites and investigate whether these metabolites are fungal antagonists.

Of the three fungi, *A. niger* showed the strongest response to mixed cultivation on straw with regard to induction of secondary metabolite gene clusters. Expression of these clusters is influenced by developmental and environmental factors (Brakhage, 2013, Keller et al., 2005), a number of which may differ between mono and mixed *A. niger* cultures. However in our study, their expression suggests they are directing antagonism towards or responding to antagonism from *P. chrysogenum* and/or *T. reesei*. For example, carbon limitation, autolysis and sporulation in *A. niger* may be increased in mixed cultures with *T. reesei* based on expression of *A. niger* cell wall modifying genes and a decrease in *A. niger* fungal biomass. Cross-referencing with expression data of *A. niger* under carbon starvation and sporulation conditions (Nitsche et al., 2012), showed that for all except one of the *A. niger* genes (encoding secondary metabolite backbone synthesising enzymes) that were induced only in mixed cultures, these conditions alone were not enough to induce expression. For *T. reesei*, it is tempting to speculate that the co-location of the secondary metabolite clusters with CAZyme encoding genes (Martinez et al., 2008) could be contributing to the lack of induction of these secondary metabolite clusters in mixed cultures. The clusters could be co-regulated with CAZyme encoding genes which decrease in transcript abundance in mixed cultures.

## 4 Conclusions

There is clear evidence for fungal interactions in the mixed cultures with wheat straw as evidenced by the changes in transcript abundance, biomass ratios and enzymatic activities. These interactions showed inter-species antagonism prevented any overall improvement in CAZyme transcript abundance or activities. *T. reesei* is the main cellulase enzyme producer used by industry but in our study it antagonised the other fungi. Although it is worth noting that in industrial production strains, unlike the wild-type strains used in this study, the effects could be different as carbon sensing and secretion levels differ. As well as to industrial strains of the species used in our study, the approaches used here could be applied to mixtures of other fungal species, such as a lignin-degrading white rot basidiomycete as well as other feedstocks or pre-treated feedstocks. Furthermore, a broader temporal profiling could uncover other fungal interactions. Our approach may not lead directly to improved enzymatic cocktails due to antagonism but could uncover transcriptional changes in each fungus in a mixed culture to better understand how CAZyme gene expression is regulated when fungi interact. Also, the orthologue analysis is a resource that could be used to investigate whether the regulation of orthologues differs in each species in response to the same experimental conditions.

## Figures and Tables

**Supplementary Figure S9:**
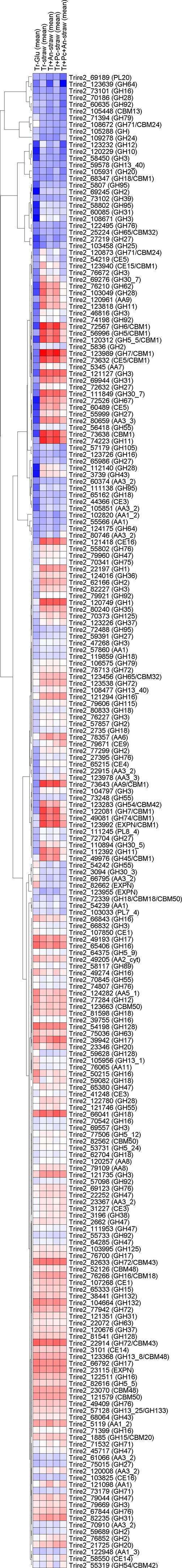
High resolution image of the hierarchical clustering of the *T. reesei* CAZy genes from Fig.8(A).

## Appendix A Supplementary material

Supplementary Table S1 List of primers and probes used. Note the format that the probes are written in indicates which nucleotides are locked nucleic acids (LNA) with upper case letter indicating an LNA. The notes section includes information on the specificity of the primer and probe combinations for discrimination between 5.8S rRNA from each species.

Supplementary Table S2 Annotations, FPKM and statistcal analysis data file. (A) List of the numbering system used for the samples and conditions in the workbook and (B) *A. niger*, (C) *T. reesei* and (D) *P. chrysogenum* CAZy and SignalP annotations, Orthofinder orthologue group composition, FPKM values as well as DESeq fold changes and significance values for listed comparisons. The members of the clusters described in Fig. 7, Fig. 8 are also listed in the respective species sheets in this file.

Supplementary Table S3 Analysis of CAZy gene contents from *A. niger*, *T. reesei* and *P. chrysogenum*. (A) Overview of the species composition of the orthogroups from the Orthofinder analysis from the three species, (B). Venn diagram comparing the CAZy families present in each of the three species, (C) total number of proteins from each species in orthogroups that contain a particular CAZy family and (D) lists of the individual orthogroups from each CAZy family. The number of proteins from each species in the orthogroup is listed along with the names of the individual proteins that make up the orthogroup with their CAZy annotation (if so annotated) in parenthesis. For *A. niger*, the activity annotations were taken from Benoit et al. (2015) and for *T. reesei*, the activity annotations were taken from Hakkinen et al. (2012).

Supplementary Table S4 Summary of the reads that were aligned uniquely to each of the genomes from the mono and mixed species cultures.

Supplementary Table S5 Full lists of enriched GO terms from the DE genes from the straw monocultures compared with either the respective glucose control or any mixed species cultures that also contain that species. The genes responsible for the enrichment of a particular GO term from a list of DE genes are indicated. The file includes a summary sheet of the enrichment pattern of any enriched GO terms.

Supplementary Table S6 Lists of CAZy genes with increased transcript abundance in mono or mixed species straw cultures compared to the glucose control and Venn diagrams comparing these lists. Any CAZy annotation except GT was used where FPKM > 1 as well as a ⩾2 fold increase in transcript abundance (DESeq counts) in the straw condition and a *p*_adj_ value < 0.05. The CAZy genes in different parts of the Venn diagram are also listed in Supplementary Table S2.

Supplementary Table S7 Induced genes indicative of competitive or antagonistic interactions in the mixed cultures from analysis of InterPro annotations of the 50 most highly induced genes in mixed cultures (*p*_adj_ < 0.05 and FPKM > 10 in mixed culture) compared to respective straw monocultures. (A) Venn diagram summarising the induced genes relevant to these interactions which are listed in (B) for *A. niger*, (C) for *T. reesei* and (D) for *P. chrysogenum*.

Supplementary Table S8 Data file related to the genes described in the section on the fungal cell wall.

Supplementary Table S9 Data file related to the genes described in the section on secondary metabolism.

Supplementary Figure S1 Relative gene expression of genes encoding for cellobiohydrolases in *A. niger*, *T. reesei* and *P. chrysogenum*. The expression level for a gene in a fungus is shown as relative to the expression level in one of the glucose control replicates for the same gene in the same fungus. The error bars in these graphs represent the standard errors from two replicate flasks.

Supplementary Figure S2 Monosaccharides quantified in the shake-flask cultures at 24 h. Error bars represent standard errors.

Supplementary Figure S3 Quantification of 5.8S rRNA from each fungus from the glucose cultures and after 24 h cultured in straw. The quantity of 5.8S rRNA was used to indicate the quantity of total RNA present. The error bars represent standard errors.

Supplementary Figure S4 Prediction of mycelial dry weight using qPCR assay of gDNA from (A) *T. reesei*, (B) *A. niger* and (C) *P. chrysogenum*. The error bars represent standard errors (n = 3). Bars with the same letters are not statistically significantly different (Tukey’s post-hoc test after ANOVA).

Supplementary Figure S5 (A) Monosaccharides released from saccharification reactions using equivalent volumes of the cultures filtrates expressed per mycelial dry weight of the culture and (B) protein concentrations in the culture filtrates expressed per mycelial dry weight of the culture. Error bars represent standard errors. Bars in a chart that contain the same letter were not significantly different (Tukey’s post-hoc test (*p* < 0.05) after ANOVA analysis).

Supplementary Figure S6 Correlation between the numbers of reads from dual RNA-seq and the copy number of the 5.8S rRNA region from each species from the mixed culture RNA samples.

Supplementary Figure S7 Validation of the transcript abundance trends as measured by FPKM from RNA-seq using qRT-PCR for a selection of genes. For each gene, the FPKM values measured by RNA-seq, the fold changes relative to the glucose control measured by qRT-PCR and the correlation between the two measures are shown. (A) *A. niger cbhA*, (B) *A. niger cbhB*, (C) *T. reesei cbh1*, (D) *T. reesei swo1*, (E) *P. chrysogenum Pc20g01970* and (F) *P. chrysogenum Pc18g05490*. The error bars represent standard errors from three replicates.
