# Supplementary material for: Transcriptomic responses of mixed cultures of ascomycete fungi to lignocellulose using dual RNA-seq reveal inter-species antagonism and limited beneficial effects on CAZyme expression
Source: Fungal Genet Biol. 2017 May;102:4–21. doi: 10.1016/j.fgb.2016.04.005 (PMC5476202; doi:10.1016/j.fgb.2016.04.005)
Supplement: Supplementary Table S1 — List of primers and probes used. Note the format that the probes are written in indicates which nucleotides are locked nucleic acids (LNA) with upper case letter indicating an LNA. The notes section includes information on the specificity of the primer and probe combinations for discrimination between 5.8S rRNA from each species. [file mmc1.pdf]

purpose	primer or probe name	sequence 5’-3’	concentration	notes[1]
quantification of *T. reesei* 5.8S rRNA	Tr_5.8S_Fw	GCGATAAGTAATGTGAATTGCAGAA	0.2µM	Primers amplify strong band from *A. niger* but do not amplify from *P. chrysogenum*. No effect on accuracy of *T. reesei* 5.8S quantification for as low as 3,200 copies when 2M copies of *A. niger* 5.8S present.
	Tr_5.8S_Rv	AATACTGGCGGGCGCAATG	0.2µM	
	Tr_5.8S_probe	atcaTcGaAtCtTtGaacg	0.1µM	
quantification of *A.*. *niger* 5.8S rRNA	An_5.8S_Fw	CAACAATGGATCTCTTGGTTCC	0.2µM	Primers amplify strong band from *P. chrysogenum* but do not amplify from *T. reesei*. No effect on accuracy of *A. niger* 5.8S quantification for as low as 80,000 copies when 2M copies of *P. chrysogenum* 5.8S present.
	An_5.8S_Rv	GCATGCCCCCCGGAATACCA	0.2µM	
	An_5.8S_probe	ttcAcAtTaGtTaTcgcat	0.1µM	
quantification of *P. chrysogenum* 5.8S rRNA	Pc_5.8S_Fw	CAACAACGGATCTCTTGGTTCC	0.2µM	Primers amplify strong band from *A. niger* and weakly amplify from *T. reesei*. No effect on accuracy of *P. chrysogenum* 5.8S quantification for as low as 3,200 copies when 2M copies of *T. reesei* 5.8S present or for as low as 80, 000 copies when 2M copies of *A. niger* 5.8S present.
	Pc_5.8S_Rv	CCCGGAATACCAGGGGGC	0.2µM	
	Pc_5.8S_probe	tcaCaTtAcGtAtCgcatt	0.1µM	
quantification of single copy region from *A. niger* gDNA in the region of the gene Aspni7_TID_1013899	Aspni7_TID_1013899_Fw	CCTCCTCATATCCGCTGTG	0.2µM	
	Aspni7_TID_1013899_Rv	GTGCTAACATGACGGGTAATTC	0.2µM	
	Aspni7_TID_1013899_probe	tccTtcCtcCtgGcttga	0.1µM	
quantification of single copy region from *T. reesei* gDNA in the region of the gene Trire2_44504	Trire2_44504_Fw	CGTTGTGCTCTCAGTGATG	0.2µM	
	Trire2_44504_Rv	GAGTCTCTGGAGTTCAGGAA	0.2µM	
	Trire2_44504_probe	acaAcgCcaTcaGcctc	0.1µM	
quantification of single copy region from *P. chrysogenum* gDNA in the region of the gene Pc20g11630.	Pc20g11630_ Fw	GCTTCTGGCTTGTGTTGG	0.2µM	
	Pc20g11630_ Rv	GGTAGTTGTGGAGCGAGATG	0.2µM	
	Pc20g11630_ probe	ttcCatCtgCcgTcgc	0.1µM	

purpose	primer or probe name	sequence 5’-3’	concen tration	notes[1]
cloning the rRNA region from any of three species into plasmid	HindIII-ITS1	**atgcatAAGCTT**TCCGTAGGTGAACCTG CGG		These primers sequences are based on publically available primer sequences - (White et al., 1990).
	SacI-ITS4-R	**atgcatGAGCTC**TCCTCCGCTTATTGAT ATGC		
measurement of expression of *T. reesei cbh1*	Trire2_123989_Fw	GCTACGATGGCAACACTTG	0.2μM	
	Trire2_123989_Rv	CCGTCCAGACAGCAGTTC	0.2μM	
	Trire2_123989_probe	cctGacAacGagAcctgcg	0.1μM	
measurement of *T. reesei swo1*	Trire2_123992_Fw	CTTCCTCTAGCTTGGTTTCAC	0.2μM	
	Trire2_123992_Rv	GCTGTATCTGTGGTTGTGTAG	0.2μM	
	Trire2_123992_probe	tcgTcaGcaTccTcatccg	0.1μM	
measurement of expression of *A. niger cbhA*	Aspni7_TID_1164901_Rv	GTAGCGATCATCCGAGTAGG	0.2μM	
	Aspni7_TID_1164901_Fw	TCTCGACCGCATTGACAC	0.2μM	
	Aspni7_TID_1164901_probe	cagCagCgaAcaGaccat	0.1μM	
measurement of expression of *A. niger cbhB*	Aspni7_TID _1117992_Fw	GTCGTCACCCAGTTCATCA	0.2μM	
	Aspni7_TID _1117992_Rv	GCCGTTGACACTGGAGTA	0.2μM	
	Aspni7_TID _1117992_probe	tgaCggCacCtcCtcc	0.1μM	
measurement of expression of *P. chrysogenum* Pc18g05490	Pc18g05490_Fw	GGAACTGGCTACTGTGACTC	0.2μM	
	Pc18g05490_Rv	CCATCTCAGGGCAGCAAG	0.2μM	
	Pc18g05490_probe	ccaCcaAcaCcaGcgt	0.1μM	
measurement of expression of *P. chrysogenum* Pc20g01970	Pc20g01970 Fw	CCGGTGCACTGGAGATTC	0.2μM	
	Pc20g01970 Rv	GCGATATGGGTTGAAATCACATC	0.2μM	
	Pc20g01970 probe	tggCggCacAtaCagc	0.1μM	

Supplementary Table S1

References

WHITE, T. J., BRUNS, T., LEE, S. & TAYLOR, J. 1990. Amplification and direct sequencing of fungal ribosomal RNA genes for phylogenetics. *In:* INNIS, M. A. (ed.) *PCR - Protocols and Applications - A Laboratory Manual.*
