# Supplementary figures and images for: Transcriptomic responses of mixed cultures of ascomycete fungi to lignocellulose using dual RNA-seq reveal inter-species antagonism and limited beneficial effects on CAZyme expression
Source: Fungal Genet Biol. 2017 May;102:4–21. doi: 10.1016/j.fgb.2016.04.005 (PMC5476202; doi:10.1016/j.fgb.2016.04.005)

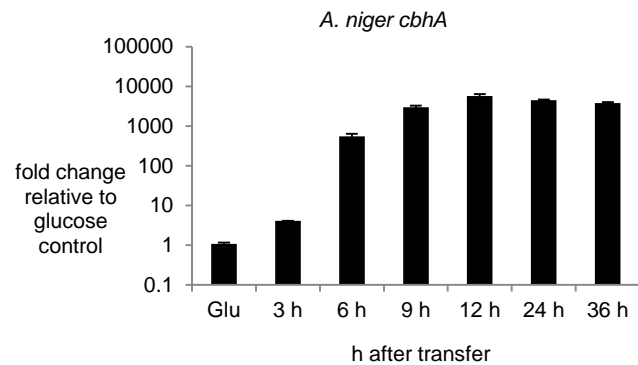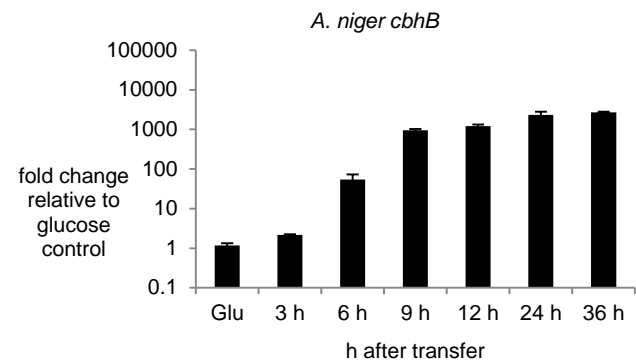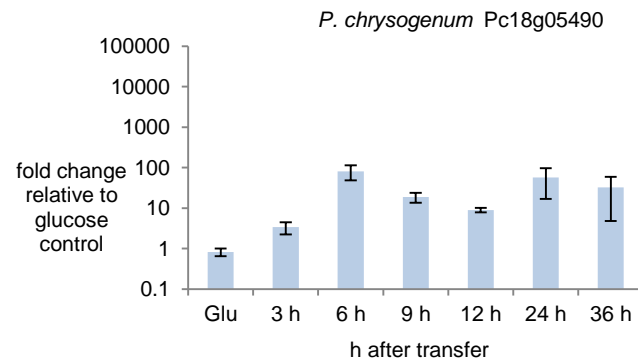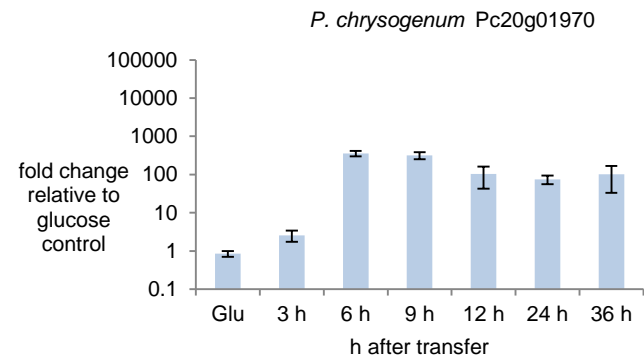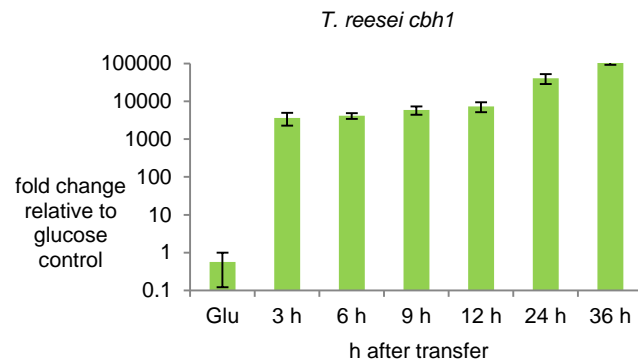

■ *A. niger*  
■ *T. reesei*  
■ *P. chrysogenum*

Supplement: Supplementary Figure S1 — Relative gene expression of genes encoding for cellobiohydrolases in A. niger, T. reesei and P. chrysogenum. The expression level for a gene in a fungus is shown as relative to the expression level in one of the glucose control replicates for the same gene in the same fungus. The error bars in these graphs represent the standard errors from two replicate flasks. [file mmc10.pdf]

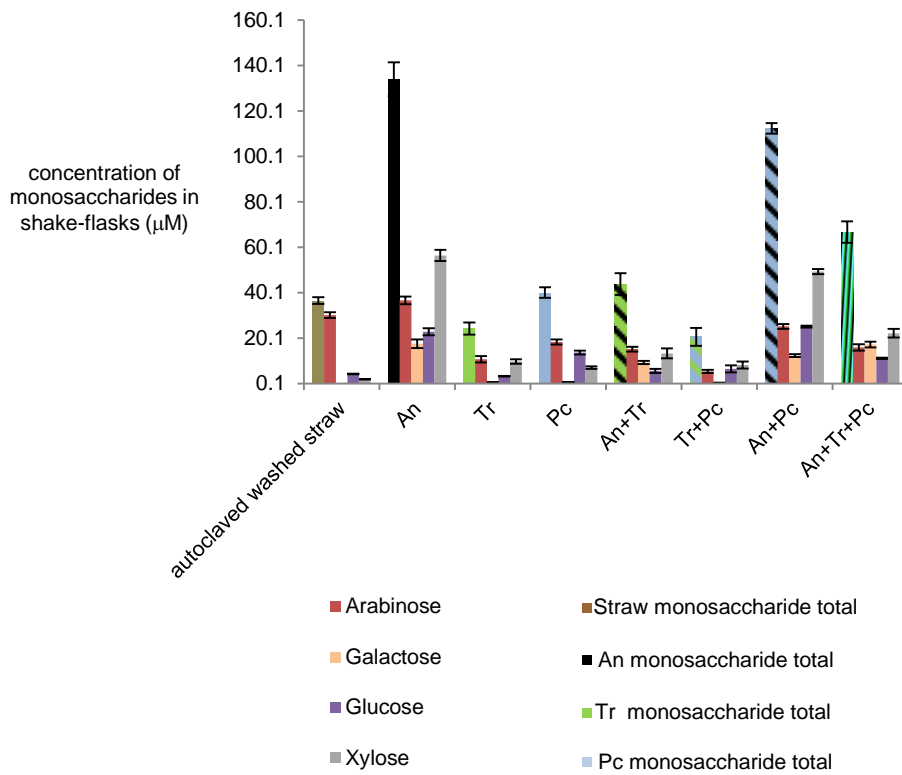

Supplement: Supplementary Figure S2 — Monosaccharides quantified in the shake-flask cultures at 24 h. Error bars represent standard errors. [file mmc11.pdf]

colour legend:  *A. niger*  *T. reesei*  *P. chrysogenum*

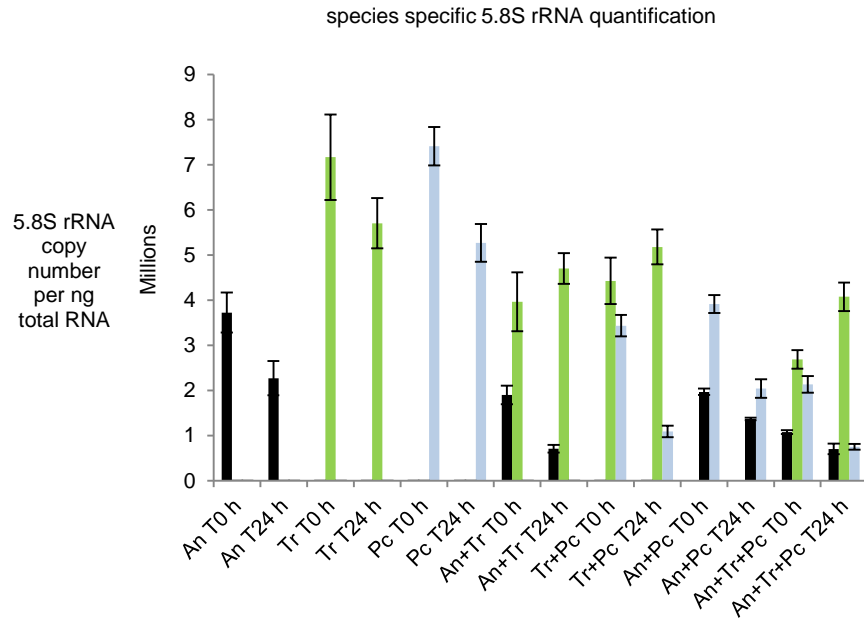

Supplement: Supplementary Figure S3 — Quantification of 5.8S rRNA from each fungus from the glucose cultures and after 24 h cultured in straw. The quantity of 5.8S rRNA was used to indicate the quantity of total RNA present. The error bars represent standard errors. [file mmc12.pdf]

(A)

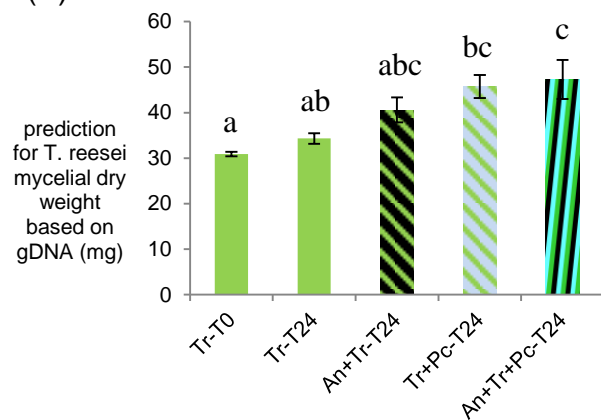

(B)

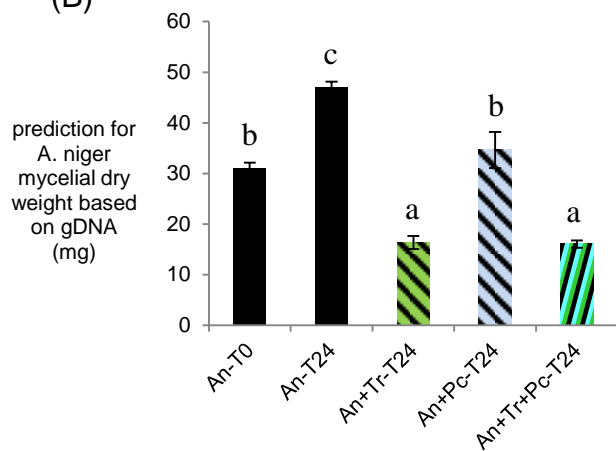

(C)

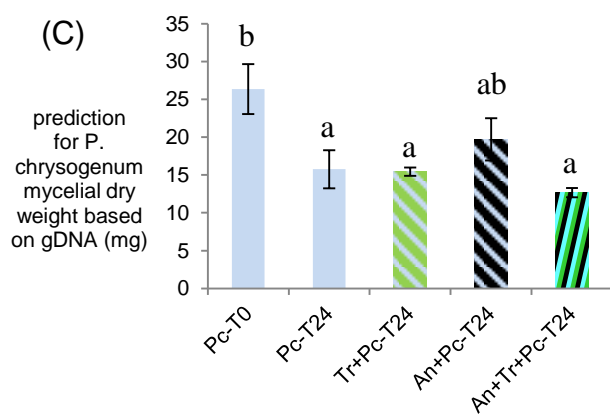

Supplement: Supplementary Figure S4 — Prediction of mycelial dry weight using qPCR assay of gDNA from (A) T. reesei, (B) A. niger and (C) P. chrysogenum. The error bars represent standard errors (n = 3). Bars with the same letters are not statistically significantly different (Tukey’s post-hoc test after ANOVA). [file mmc13.pdf]

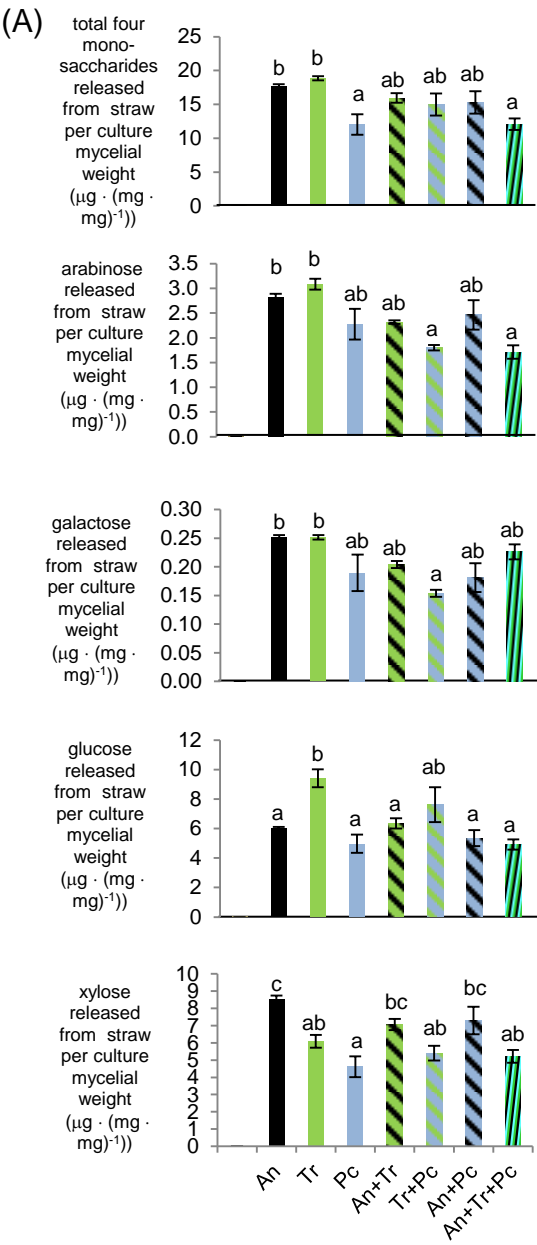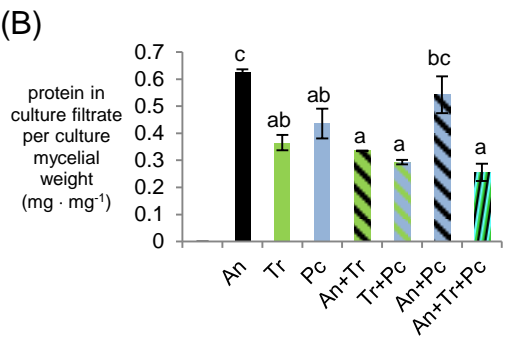

Supplement: Supplementary Figure S5 — (A) Monosaccharides released from saccharification reactions using equivalent volumes of the cultures filtrates expressed per mycelial dry weight of the culture and (B) protein concentrations in the culture filtrates expressed per mycelial dry weight of the culture. Error bars represent standard errors. Bars in a chart that contain the same letter were not significantly different (Tukey’s post-hoc test (p < 0.05) after ANOVA analysis). [file mmc14.pdf]

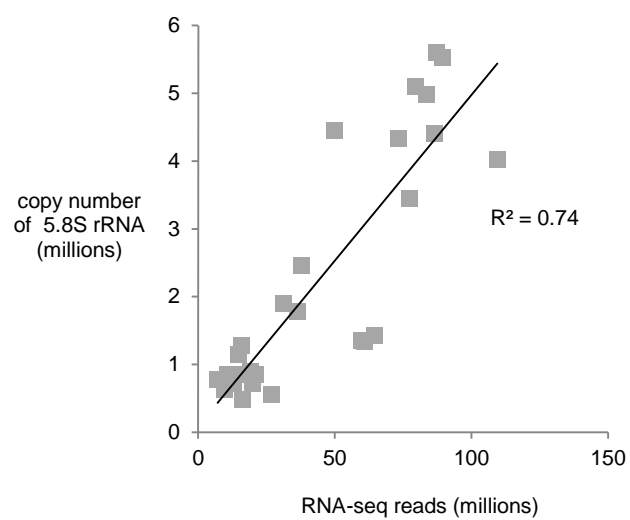

Supplement: Supplementary Figure S6 — Correlation between the numbers of reads from dual RNA-seq and the copy number of the 5.8S rRNA region from each species from the mixed culture RNA samples. [file mmc15.pdf]

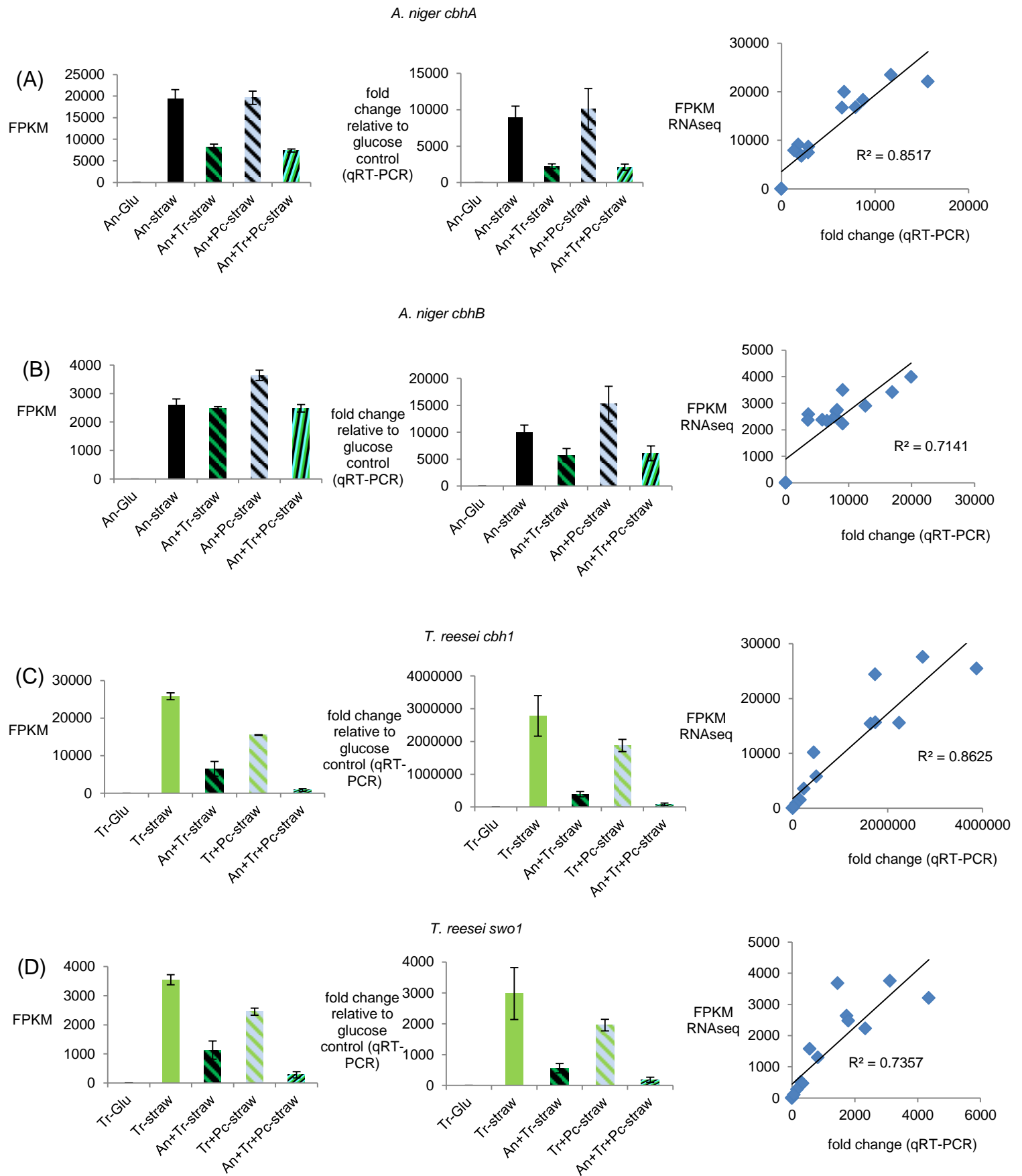

*P. chrysogenum* Pc20g01970

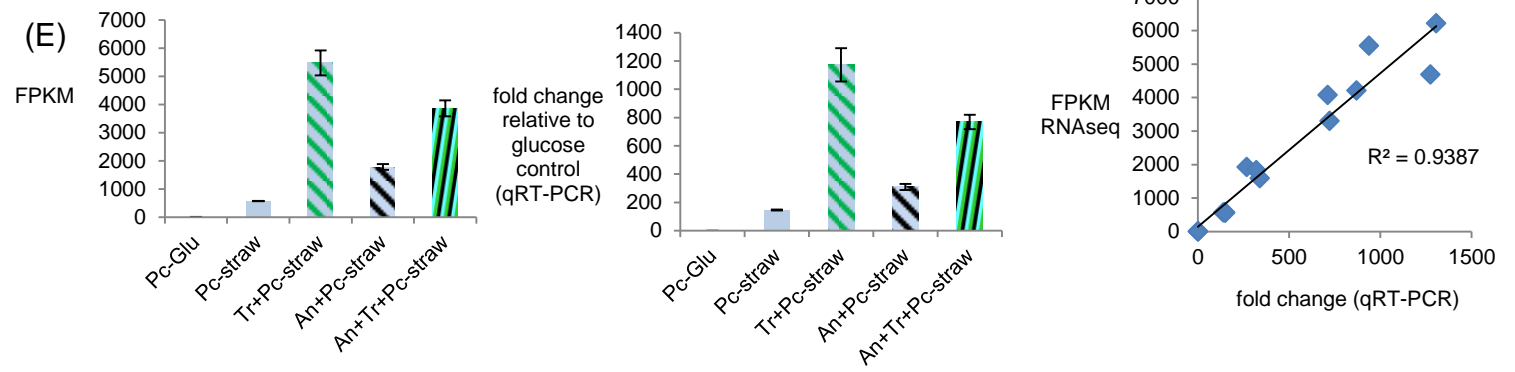

*P. chrysogenum* Pc18g05490

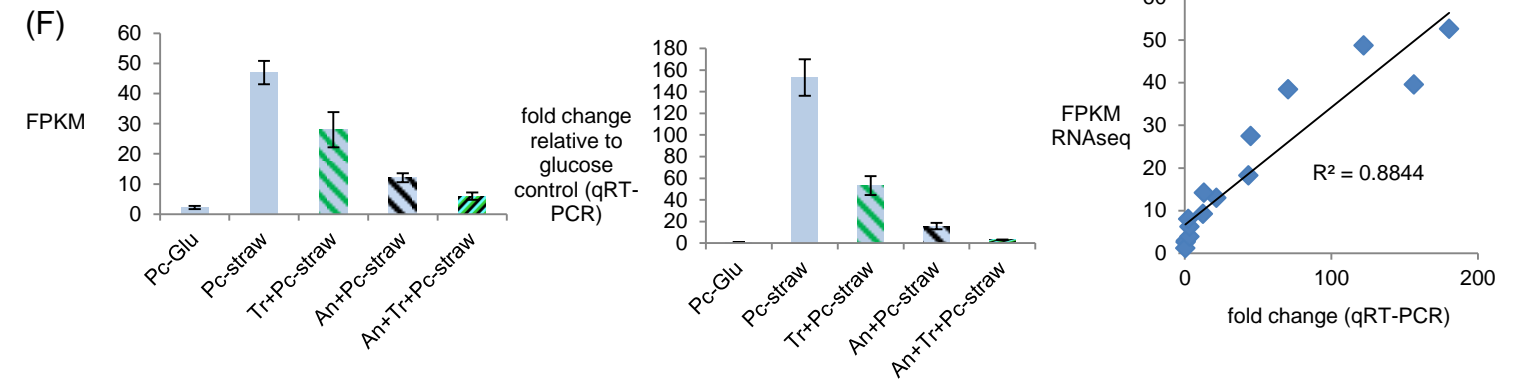

Supplement: Supplementary Figure S7 — Validation of the transcript abundance trends as measured by FPKM from RNA-seq using qRT-PCR for a selection of genes. For each gene, the FPKM values measured by RNA-seq, the fold changes relative to the glucose control measured by qRT-PCR and the correlation between the two measures are shown. (A) A. niger cbhA, (B) A. niger cbhB, (C) T. reesei cbh1, (D) T. reesei swo1, (E) P. chrysogenum Pc20g01970 and (F) P. chrysogenum Pc18g05490. The error bars represent standard errors from three replicates. [file mmc16.pdf]
